# Bone marrow lesions in osteoarthritis: Characterising genetic and histological changes to understand disease pathophysiology

**DOI:** 10.1016/j.ocarto.2024.100531

**Published:** 2024-10-26

**Authors:** Nidhi Sofat, Franklyn Arron Howe

**Affiliations:** a Institute for Infection and Immunity, School of Health & Medical Sciences, City St Georgeʼs, University of London, London, UK; b Department of Rheumatology, St George’s University Hospitals NHS Foundation Trust, UK; c Neuroscience & Cell Biology Research Institute, School of Health & Medical Sciences, City St Georgeʼs, University of London, London, UK

**Keywords:** Osteoarthritis, Bone marrow lesions, Osteoarthritis bone score, Genetics

## Abstract

Osteoarthritis (OA) is a chronic debilitating condition that affects the whole joint. There are several sources of pain in OA that include the synovium, bone, including osteophytes and more recently bone marrow lesions (BML) that correlate with pain. Recent studies have shown that the bone compartment contributes to pain in OA through the development of OA-BMLs which are richly innervated and demonstrate angiogenesis. The synovium is also innervated in OA tissue and is another distinct source of pain, with imaging and genetic studies supporting the observation that synovitis is an important component of pain in OA. Previous studies using magnetic resonance imaging (MRI) have shown that bone marrow lesions (BMLs), observed as high intensity signal on T2 fat-suppressed imaging sequences, are commonly found in OA and are associated with progression of pain symptoms. Recent studies have described the genetic signature of BMLs and the characteristic histological changes of BML tissue. In this narrative review we describe the recent developments in the discovery of the gene expression profiles identified from BMLs. We also review the recently characterised histological changes from BMLs in large weight-bearing joints including the knee and hip. Finally, we discuss the implications of new genetic and histological findings in BML in the context of new developments for pharmacological therapies in OA.

## 1 Introduction

Osteoarthritis (OA) is a common condition affecting millions of people globally [1]. Despite its high prevalence there are currently no licensed disease-modifying drugs that halt disease progression [2,3]. Numerous large-scale clinical trials have been conducted to test new pharmacological therapies in OA, particularly for knee and hip involvement, but these have not led to the approval of new treatments for this debilitating condition. Historically, the field has focused on regeneration of cartilage in attempts to achieve clinically meaningful improvements in pain and function after intervention. For example, recently the FORWARD study tested the effect of the anabolic agent, sprifermin, a recombinant protein consisting of fibroblast growth factor-18 (FGF-18), on cartilage thickness, volume and pain outcomes in OA [4]. Although there was an improvement in cartilage regeneration thickness component of the knee joint with sprifermin, it failed to reach a clinically meaningful effect for pain and stiffness [4]. Further clinical trials in large joint OA have targeted pain modulation and have included novel therapeutic agents, including monoclonal antibodies to nerve growth factor e.g. tanezumab [5,6]. However, following Phase III clinical trials with tanezumab, licensing has not progressed further due to concerns about rapidly progressive OA in a subgroup of people with knee OA.

Participants with OA in clinical studies may also demonstrate variation in the individual effectiveness of treatment. People with OA are left not knowing if a treatment will work or for how long, and when or why their symptoms get worse. Many current clinical trials in OA recruit participants with wide disease heterogeneity, resulting in a current situation where a streamlined approach does not exist for stratifying participants to specific treatments. Recent research strategy groups have proposed a multi-modal approach, using technology to assist in OA stratification that could enhance OA trial design [9]. There is an urgent need to develop better treatments for OA, since many non-surgical treatments for OA only offer short-term relief.

OA is a condition causing changes in several joint compartments, including the synovium, bone, cartilage and meniscal structures [9]. Previous studies have demonstrated that bone marrow lesions (BMLs) in OA are strongly associated with pain. One of the earliest studies of OA-BMLs, led by Felson et al. [10], reported a study of 401 knee OA participants, 50 of whom had no knee pain. Participants underwent coronal T2-weighted fat suppressed MRI scans and BMLs were graded by their size. The frequency of BMLs increased with radiographic grade of OA: 48 ​% of Kellgren- Lawrence Grade 0 had BMLs compared with 100 ​% of those with Kellgren- Lawrence Grade 4. In addition, BMLs were found in 78 ​% of the painful knee group compared with 30 ​% of the non-painful knee group (P ​< ​0.001). In another study of BMLs in the OA participants analysed by painful and non-painful OA groups, larger lesions (>1 cm^2^) were more common in the painful versus the non-painful knee OA group (P ​< ​0.05) [11]. In a study of women with knee OA [11], the participants with larger BMLs were more likely to have full-thickness cartilage defects, adjacent subcortical bone abnormalities and painful knee OA with an odds ratio of 3.2 [11].

Since its first descriptions of BML associations with pain in 2001, numerous studies using large datasets such as the Multicentre Osteoarthritis Study [12], OsteoArthritis Initiative [13] and clinical trials [14] have provided further support to the observation that OA-BMLs are an important contributor to pain. Furthermore, scoring systems to assess imaging changes characteristic of BMLs [15] have developed, including the MRI knee osteoarthritis score (MOAKS) [16] to aid further research into the pathophysiology of OA-BMLs.

Currently, many clinical trials and studies are collecting measures on structural changes in the whole OA joint e.g. by MRI, to include cartilage, bone and synovium changes in response to specific interventions [[15], [16], [17]]. In addition to cartilage and bone, the synovium can become inflamed in OA and active synovitis is a treatment target in OA. Traditional inflammatory disease modifying therapies have also been tested in hand OA e.g. hydroxychloroquine but were not found to be effective in improving pain [7], although recent work has suggested that other disease-modifying anti-rheumatic drugs such as methotrexate may be an agent that can target OA synovitis [8].

In this narrative review, we describe recent developments in our understanding of OA-BML pathophysiology and review the literature describing the genetic signature and histological profile of OA-BMLs. The implications of new developments in OA-BML pathophysiology are described in the context of novel therapies that are being developed to target OA-BML modulation as a therapy for OA.

## 2 Methods

**Fig. 1:**
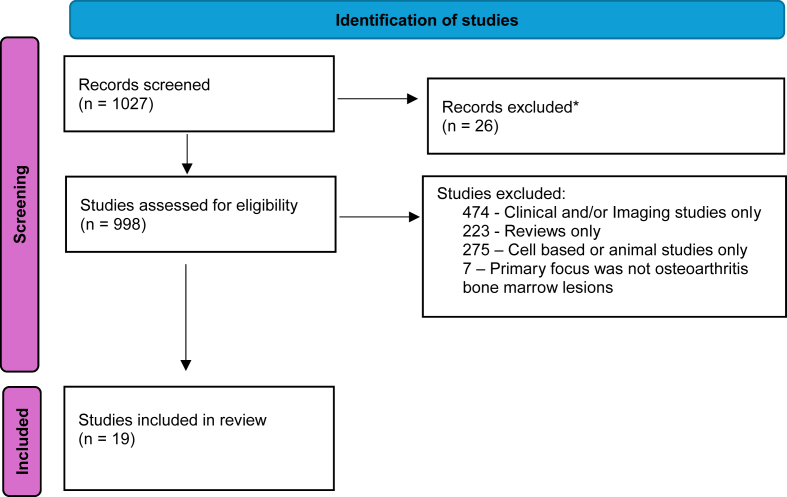
Modified Prisma Flow Diagram for study identification. *Duplicate studies, or not in English language.

A literature search was undertaken from 1 January 1990 to 1 August 2024 using electronic databases: Medline (Ovid), Embase (Ovid), Medline, Web of Science and CINAHL (EBSCO) for this narrative review. The search terms “osteoarthritis” and “bone marrow lesions” were used. Studies which reported genetic and histological studies of human OA-BMLs were identified. For the purpose of this narrative review, studies which reported clinical and/or imaging data alone were excluded. Studies in animal models were excluded. We identified a total of 1024 publications using the search terms bone marrow lesions and osteoarthritis (Fig. 1). By including additional search terms of ‘genetics’ and ‘histology’, a total of 19 publications were identified, which are the subject of this narrative review. Review articles and animal models were excluded. Original articles reporting data in human OA were then assessed and reported in this narrative review.

### 2.1 Genetic studies of OA-BMLs

**Fig. 2:**
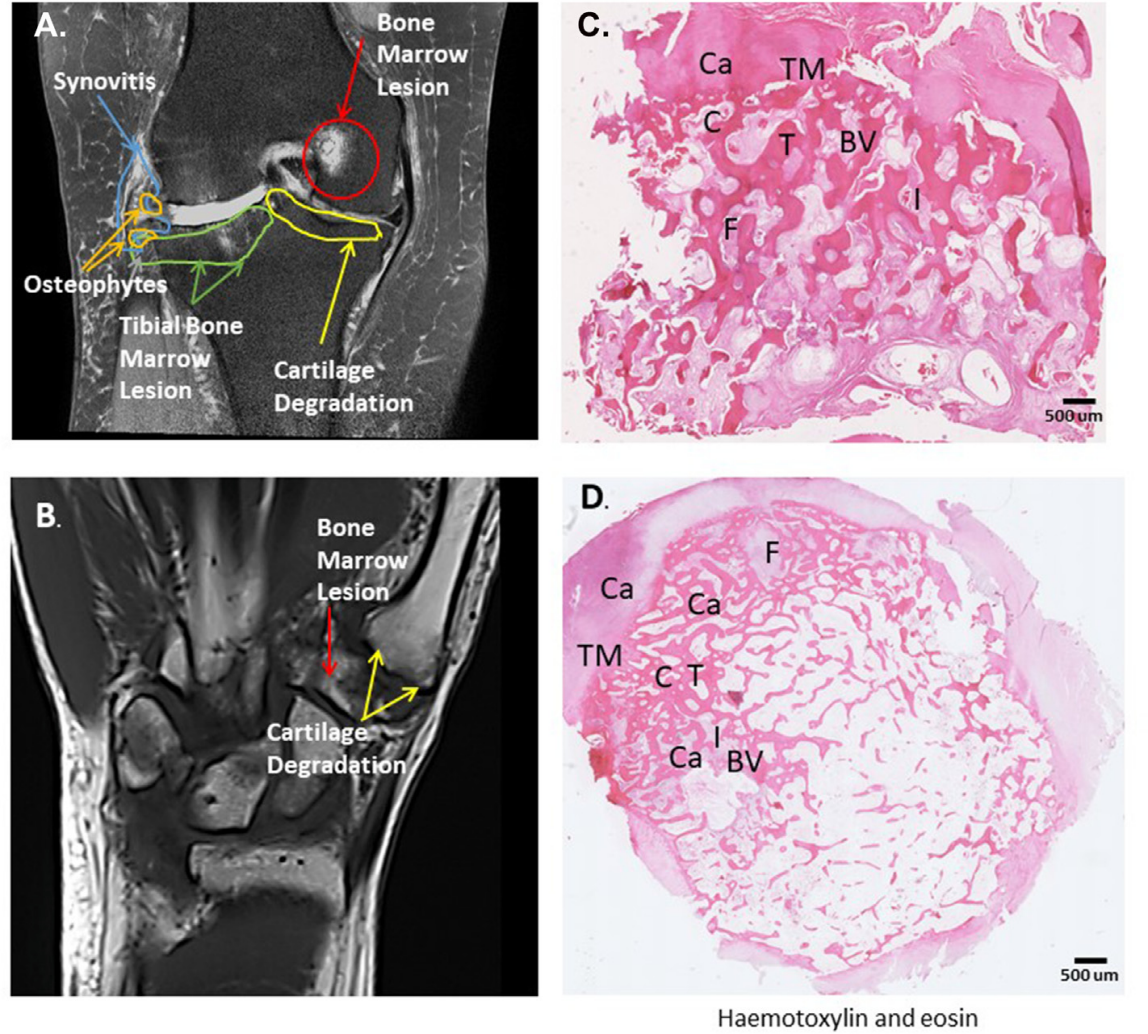
Presence of OA-BML in knee and hand OA. Results from imaging and tissue analysis in knee and hand osteoarthritis from the Pain Perception in Osteoarthritis study (the study was conducted with full Ethical Approval, Research Ethics Committee approval number 12/LO/1970). A. Magnetic Resonance Imaging (MRI) scan of knee of participant with osteoarthritis demonstrating osteophytes, synovitis, cartilage degradation and bone marrow lesions. B. Magnetic Resonance Imaging (MRI) scan of hand participant with osteoarthritis demonstrating cartilage degradation and bone marrow lesions. C. Histological section of medial tibial subchondral knee tissue from participant with OA-BML. D. Trapezium bone from participant with hand OA undergoing trapeziectomy. The 7 typical features showing OA-BML changes include Cysts (C), Fibrosis (F), blood vessels (BV), Thickened trabeculae (T), cartilage (Ca), tidemark integrity (TM), inflammation (I) which comprise the Osteoarthritis Bone Score (OABS).

**Fig. 3:**
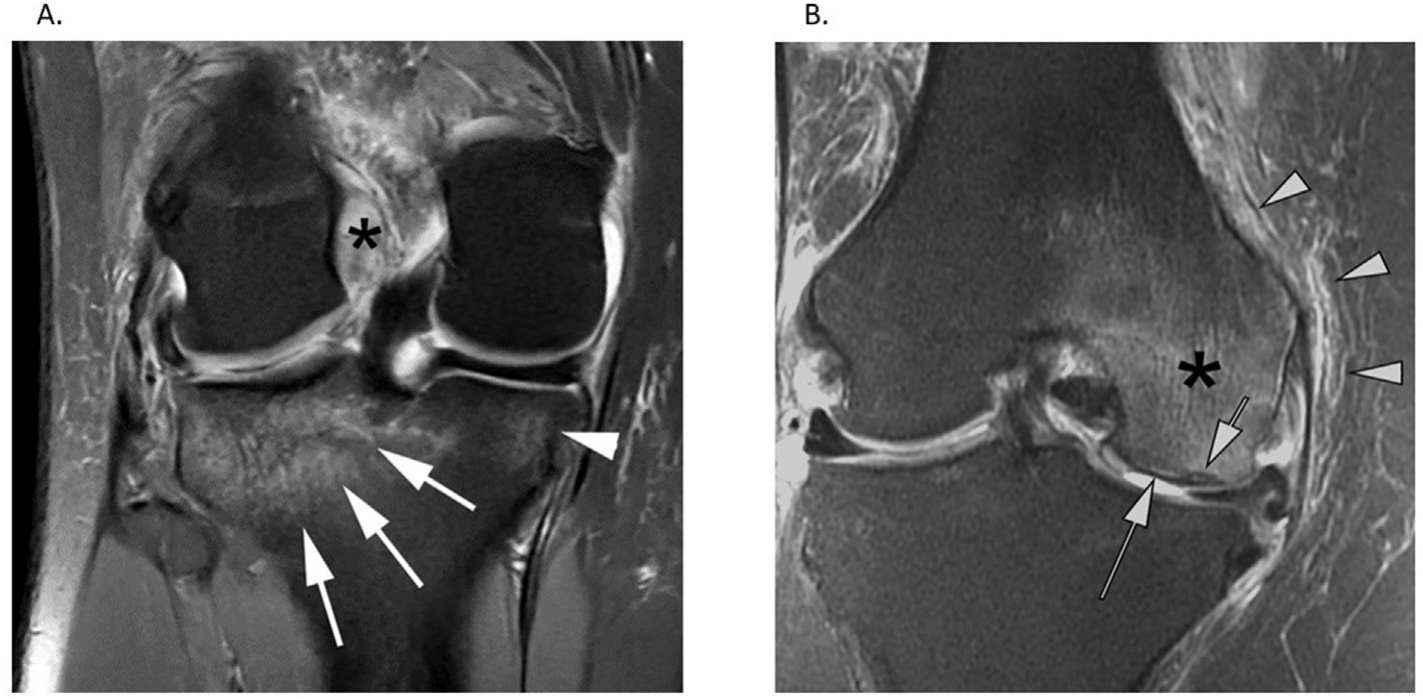
Bone marrow lesions caused by alternative pathologies to OA. A. BML caused by trauma: Coronal fat-suppressed intermediate-weighted MRI shows hyperintensity of the posterior lateral tibial plateau, but there is no fracture line (arrows). There is also a smaller hyperintense lesion is visible at the posterior medial tibia (arrowhead). There is also a traumatic anterior cruciate ligament tear (asterisk) and the bone marrow changes are consistent with bone contusions found in association with the cruciate ligament tear. B. BML caused by Subchondral Insufficiency Fracture (SIF). Coronal fat-suppressed MRI shows a subchondral linear hypo-intensity zone directly adjacent to the normal subchondral plate (short arrow) at the medial femoral condyle. There is also extensive bone marrow hyperintensity of the femoral condyle (‘bone marrow oedema’, asterisk) and soft tissue hyperintensity (‘inflammation’) at the medial joint line (arrowheads). Subchondral linear hypo-intensity is pathognomonic for SIF. There is also full-thickness cartilage loss at the central medial femur (long arrow) and meniscal extrusion due to a posterior medial meniscus root tear, which are commonly found with SIF.

OA-BMLs are characterized by hypo-intensity on T1 weighted images, and hyperintensity on T2, proton density-, and intermediate-weighted fat-suppressed fast and short tau inversion recovery MRI sequences (for example see Fig. 2). OA-BMLs are most frequently observed where they are adjacent to fibrillated and denuded articular cartilage in the subchondral compartment, without any visible fracture line. It is important to exclude other causes of bone marrow oedema in studies, including BMLs representing trauma, subchondral insufficiency fracture or malignancy (Fig. 3).

Several studies have used cell and tissue extraction techniques from BMLs to identify the genetic transcriptomic signature of BMLs. Three large studies to date have identified the transcriptome from OA-BMLs [[17], [18], [19]] (Table 1). In the first transcriptomic study of OA-BMLs, Kuttapitiya et al. [17] found that 218 genes were upregulated in human knee OA-BML compared to healthy non-OA bone. The most upregulated genes included stathmin 2, thrombospondin 4, matrix metalloproteinase 13 and Wnt/Notch/catenin/chemokine signalling molecules that are known to constitute neuronal, osteogenic and chondrogenic pathways [17]. Tuerlings et al. [18] performed RNA sequencing on macroscopically preserved and lesional OA subchondral bone from patients with OA hip or knee. They identified 1569 genes that were significantly differentially expressed between lesional and preserved subchondral bone, including CNTNAP2 and STMN2. Among these 1569 genes, 305 were also differentially expressed, and with the same direction of effect, in cartilage, including the recently recognized OA susceptibility genes IL-11 and CHADL. Specific genes were differentially expressed in subchondral bone of the knee, including KLF11 and WNT4. Zeng et al. also reported upregulation of IL-11 and VCAN from knee OA BMLS [19], supporting the role of IL-11 in OA-BML pathophysiology.

**Table 1:** Gene pathways upregulated in bone marrow lesions.

**Neuronal pathway genes:** Stathmin 2 (STMN2) Thrombospondin 4 (THBS4) Neuronal tyrosine-phosphorylated phosphoinositide-3-kinase adaptor 2 (NYAP2)	**Angiogenesis signalling pathways:** Vascular epidermal growth factor (VEGF) Nuclear factor kappa B (NF-κB) Interleukin-11 (IL-11)
**Chondrocyte-based genes:** Matrix metallopeptidase 13 (MMP-13) Collagen type XVI CHADL (chondroadherin-like)	**Bone turnover genes:** Catenin delta (CTNND2) Homeobox 1 and 2 RANK Ligand (RANKL)

A summary of the gene expression upregulated in human OA-BMLs (see Refs. [[17], [18], [19]]).

### 2.2 Characterisation of histological changes in OA-BMLs

Although a large proportion of people with OA receive medical management, in cases where joint surgery is required due to intractable pain symptoms e.g. hip, knee or hand, the tissue harvested at joint surgery is a rich source of information which has increased our understanding of OA-BML pathology. Samples from joint surgeries have demonstrated features of angiogenesis and new nerve formation [20,21]. Previous work has been conducted on BMLs in distinct anatomical sites, including the hand, knee and hip. Taljanovic et al. [22] showed that BMLs in hip OA can be observed clearly by MRI scan before joint replacement surgery and correlated with histological changes that includes cysts, pseudocysts and microfractures represented by areas of osteoclast activity and angiogenesis in the subchondral bone.

Koushesh et al. [23] demonstrated that knee OA-BMLs also have very similar histological changes to hip OA-BMLs from tissue harvested at joint replacement surgery [22]. OA-BMLs are associated with structural change, including lost osteochondral integrity, fibrosis, cysts, and de novo cartilage within subchondral bone. While non-BML regions of OA subchondral bone display bone attrition, BMLs display trabecular thickening (but with reduced mineralisation) consistent with high turnover. Increased vascularity and perivascular innervation in BMLs might contribute to pain and are a consistent feature of OA-BMLs [23]. Koushesh et al. [23] demonstrated that hypervascularity in BML tissue is most frequently observed near the osteochondral junction, with other regions of increased blood vessels deep within the subchondral bone. Subchondral vascularity was higher in BML tissue 123.5 (SD 69.1) compared with non-BML tissue 53.2 (SD 21.4) and post-mortem controls 11.7 (SD 5.4) p ​< ​0.0001 [23]. Staining for nerves with PGP9.5 immunoreactive nerve profiles was also most frequently observed in a perivascular distribution at the osteochondral junction and deeper within subchondral bone [23].

More recently, BMLs have also been detected in people with hand OA in the trapezium bone for people undergoing surgery for hand OA [24]. People with hand OA who had already received full medical management, including non-steroidal anti-inflammatory drugs, intra-articular steroid injections and hand therapy, underwent trapeziectomy for hand OA [24]. Pre-operatively, MRI-defined changes using the OMERACT thumb base scoring system found the presence of cartilage damage, subchondral changes and bone marrow lesions (Fig. 2). Changes on MRI were able to colocalise changes correlating to BMLs from harvested tissue. The OABS was applied to all trapeziectomy tissue samples, with scores ranging from 6 to 7 in all the samples evaluated [24]. Assessment of pain sensitisation using painDETECT showed significant correlation to the summed the OMERACT thumb base scoring system for: number of subchondral bone defects (R ​= ​0.66, p ​= ​0.007), number of osteophytes (R ​= ​0.72, p ​= ​0.002) and cartilage degradation (0.56, p ​= ​0.031). A practical guide to assess BMLs can assist research groups in evaluating OA tissue for BMLs (the OABS training manual for interpreting OA tissue sections is provided in the supplementary information to this review).

Studies from hip, knee and hand OA demonstrate changes which are found commonly in all three anatomical sites. Despite differences in anatomy, joint loading, weight bearing, specific risk factors e.g. menopause in hand OA, the presence of BMLs in joints as diverse as the hand, knee and hip show that OA-BMLs are likely to represent a shared pathway of joint damage that is found in OA. As such, OA-BML represent an attractive therapeutic target for OA treatment.

Recently, significant progress has been made in other fields of medicine by identifying the clinical, histopathological and genetic correlates of disease. For example, in oncology, a tissue biopsy of a malignant lesion can be phenotyped for clinical features, histopathological changes and genetic signatures [25]. By obtaining detailed ‘mapping’ of e.g. a tumour’s characteristics, predictions can be made based on gene and protein characteristics for treatment choice, responsiveness and prognosis. There is now increased recognition that OA has several phenotypes [26], but information about clinical correlates of structural damage, gene and protein signatures are less well characterised in OA. Attempts have been made in other rheumatic diseases, including synovial tissue changes in rheumatoid arthritis, which assist in disease stratification and treatment consideration options [27]. By characterising the specific gene and protein signatures of OA including cartilage, synovium and bone we can understand the histopathological changes which contribute to the OA disease process.

### 2.3 How can we measure BML changes?

The quantification of OA-BMLs is an important step if we are to show that interventions can target and modify BMLs. OA-BMLs can be assessed semi-quantitatively with the MRI OA Knee Score (MOAKS) [13] or Rapid OA MRI Eligibility Score [28]. MOAKS includes detailed subregional grading of areas of presumed BML together with associated cysts containing fluid equivalent signal directly adjacent to the subchondral plate. MOAKS has been used in several clinical trials and epidemiological studies [13,29,30]. Rapid OA MRI Eligibility Score is a simplified measure used for defining structural eligibility of participants for inclusion in clinical trials. Quantitative measurement of BMLs using image segmentation can also be performed using scores such as the Knee Inflammation MRI Scoring System [31].

More recently, artificial intelligence methods have been used to provide a more rapid assessment of MRI changes to identify specific changes. For example, AI-assisted MRI has been used to acquire image sequences more rapidly while not compromising image quality [32]. Some protocols have reduced scanning time but maintained image quality [33] and machine learning tools are being developed to assist in automation of scoring systems which may assist in MRI scoring of lesions in the future [34].

BMLs have been identified in animal OA models, where histological measurement might have advantages due to the small size of the rodent joints often used for preclinical testing of novel pharmacological agents [35] which can then be applied to clinical trials using OA-BMLs as a readout. The recently described OA Bone Score (OABS) [23], grades 7 BML-associated histopathological characteristics, and, like MRI scoring systems such as MOAKS and ROAMES, displays good reliability. The OABS identifies characteristic histological changes in OA-BML, including cysts, fibrosis, disruption of tidemark integrity, new blood vessel formation, fibrosis, inflammatory infiltrates and thickened trabeculae in subchondral bone (Table 2). The OABS effectively discriminated between OA and non-OA medial tibial osteochondral samples and was better able to distinguish BML from non-BML bone than the Mankin’s chondropathy grade [23]. Further analysis of the distinct histological processes within BMLs using a Rasch analysis from the same study showed that there are two inter-related pathological processes, affecting trabecular and non-trabecular structures respectively [23]. Future work is required to investigate the temporal sequence of OA-BMLs in relation to the histopathological signature of OA-BMLs.

**Table 2:** Scoring system OABS.

Osteoarthritis Bone Score (OABS)	Grade
**1. Cysts**	
**None**	**0**
**Present (at least 1)**	**1**
**2. Fibrosis (fibrotic connective tissue within bone marrow space)**	
**None**	**0**
**Present (at least one region)**	**1**
**3. Blood vessels (number of blood vessels within the subchondral region of interest)**	
**Normal (0–15)**	**0**
**Increased (>16)**	**1**
**4. Cartilage islands (new cartilage within bone)**	
**Absent**	**0**
**Present**	**1**
**5. Trabeculae thickened (≥2 trabeculae >200 ​μm wide)**	
**Normal**	**0**
**Increased thickness**	**1**
**6. Tidemark integrity**	
**Intact**	**0**
**Crossed by at least one blood vessel**	**1**
**7. Inflammation (cellular infiltrates)**	
**Absent**	**0**
**Present**	**1**
**Total**	**7**

Legend. The OsteoArthritis Bone Score (OABS) is characterised by the presence of 7 characteristic features summarised in the table. To score 1 in any domain, the feature described needs to be observed at least once in the OA tissue section (Ref. [23]).

### 2.4 Importance of BMLs as a therapeutic target

OA-BMLs might help to identify people at risk of symptomatic and structural OA progression who are most likely to benefit from treatment. BMLs might identify either an OA subtype or phase of disease that could benefit from specific treatment [14]. Further research is required to determine whether some individuals, perhaps with distinct genetic constitution, joint structure or OA aetiology. It is important to identify individuals at higher risk of developing BMLs, the relation to cartilage defects and synovitis, to assess or whether BMLs reflect a specific phase of OA development and progression.

The work of both Kuttapitiya et al. [17] and Tuerlings et al. [18] identified similar target OA-BML genes from their studies, including STMN2 and wnt/catenin pathway genes. The findings from the respective gene array studies open up potential new avenues for treatment e.g. stathmin 2 has been identified in several studies. Since stathmin 2 is a microtubule-associated protein that is involved in axonal development and repair, then inhibitors targeting this protein could be developed in future therapeutic studies. The wnt/catenin pathway has also been implicated in several studies and work is currently underway including wnt pathway modulators e.g. lorecivivint and more recently the anti-sclerostin antibody romosozumab is being tested in clinical studies for OA. With respect to IL-11, this pro-inflammatory cytokine is implicated in cell senescence and ageing [36]. Anti-IL-11 therapy is currently in early-stage clinical trials for fibrotic lung disease [37] and it has also been proposed as a potential therapeutic agent on pathology involving ageing, such as OA [38].

Pharmacological and non-pharmacological targeting of OA-BMLs might represent a novel treatment class to both rapidly improve symptoms, delay structural and symptom progression, and reduce the currently high need for joint replacement surgery, particularly of large weight-bearing joints [39]. Clinical trials should appreciate differential diagnoses, because some BMLs might be inappropriate for OA-BML treatment, for example BMLs representing trauma, subchondral insufficiency fracture or malignancy (Fig. 3). There have been attempts to reduce OA-BMLs which have targeted subchondral bone turnover bisphosphonates [[40], [41], [42], [43]], strontium ranelate [44]. Recently, a phase 2 trial was completed assessing the effect of pentosan polysulfate (PPS) in knee OA [48]. PPS is a potential treatment target for OA-BML and inhibits NFKB, which is upregulated in OA-BML [17]. Since PPS acts via NFKB it could act via several mechanisms in OA to reduce inflammation, pain sensitisation, cartilage degradation and improve blood flow. Results from a Phase 2 trial in knee OA demonstrated that OA-BMLs reduced in size by treatment with PPS [48].

Non-pharmacological approaches include offloading the affected joint by reducing the biomechanical stresses thought to mediate BML formation or pain, including high tibial osteotomy [45] or patellofemoral bracing [46]. Other treatments might remove or replace BMLs such as arthroplasty, or more generally restore normal cellular function (Bone Marrow Concentrate and Platelet Product injections [47]). Treatments targeting sensitising molecules such as nerve growth factor or Trk A may also reduce pain by acting on factors produced within BMLs. Other treatments that can reduce pain associated with BMLs, such as exercise, analgesics and weight loss may exert their effect without reducing BMLs [49,50].

Assessing clinical responses to BML-targeted interventions might be most expected in the subgroup of individuals for whom BMLs are the predominant cause of pain or structural disease progression. OA-BML assessment could enable OA stratification by identifying a treatment-responsive OA patient subgroup. More recently, bone modulator drugs have been suggested as modifiers of subchondral structural change in OA; a recent clinical trial of denosumab, a monoclonal antibody targeted at RANK Ligand demonstrated that in hand OA, treatment with denosumab resulted in an improvement of the primary (radiographic) endpoint, which was the change in the total Ghent University Scoring System at week 24, where positive changes correspond to remodelling and negative changes to erosive progression [51]. The primary endpoint was met with an estimated difference between groups of 8.9 (95 ​% confidence interval 1.0 to 16.9; *P* ​= ​0.024) at week 24. There were also more erosions found in the placebo group (125 events in 44 patients (90 ​%)) compared with the denosumab group (97 events in 41 patients (80 ​%)). The results from the hand OA denosumab trial suggest that it can achieve structure modification in erosive hand OA by promoting remodelling and reducing the development of new erosions. Other bone-modulating drugs e.g. romosozumab have also been tested in OA, although results of a benefit for pain in knee OA was inconclusive [52].

## 3 Concluding remarks

Recent studies have demonstrated that OA-BMLs are dynamic structures with a distinct genetic and histological profile. Genes involved in new nerve formation, angiogenesis and inflammation feature highly in OA-BMLs, with tissue changes showing increased nerve/blood vessel formation, new cartilage formation and inflammation. Further studies are needed to investigate if a treatment is more effective for or better tolerated by individuals with BMLs than those without BMLs. New interventions that target key biochemical or structural aspects of OA-BMLs [53], will assist in identifying their importance in OA and to address the high burden of symptoms caused by this condition.

## Declarations

The views expressed in this publication are those of the authors and not necessarily those of the NHS, NIHR, the Department of Health or Social Care.

## Ethical Approval

For human tissue analysis, full Ethical approval was obtained from the London Research Ethics Committee, approval number 12/LO/1970.

## Funding

We would like to acknowledge funding from The Rosetrees’ Trust (Grant number M11-F3) and St George’s Hospital Charity (Grant number LEG23010-11409) for funding this work. We gratefully acknowledge the patients who participated in our research studies.

We also acknowledge surgeons Mr Philip Mitchell, Ms Shamim Umarji, Ms Sonja Cerovic and Mr Jamil Moledina in assisting with sample collection from orthopaedic surgery at St George’s University Hospital NHS Foundation Trust. We acknowledge technical assistance from Soraya Koushesh, Seyi Taylor-Kuti and Andisheh Niakan for tissue preparation, sectioning and staining.

## Conflict of interests

The authors declare that they have no known competing financial interests or personal relationships that could have appeared to influence the work reported in this paper.
