# Supplementary material for: Bone marrow lesions in osteoarthritis: Characterising genetic and histological changes to understand disease pathophysiology
Source: Osteoarthr Cartil Open. 2024 Oct 26;6(4):100531. doi: 10.1016/j.ocarto.2024.100531 (PMC11565461; doi:10.1016/j.ocarto.2024.100531)

OABS scoring - training

BMLs

OA known as “disease of the cartilage”

Cartilage -> aneural and avascular structure, other factors must contribute to pain.

Microarray analysis on BML tissue found a no. of upregulated genes involved in neurogenesis, chondrogenesis, and inflammation (1)

BMLs are already well described in the literature by MRI and have been associated with pain (2), <u>but little is known about their histological changes</u>.

AIM: to develop a novel histological scoring system to describe BMLs by comparing BML tissue versus non-BML OA tissue and normal bone.

1) Kuttapitiya A, Assi L, Laing K, Hing C, Mitchell P, Whitley G, Harrison A, Howe FA, Ejindu V, Heron C, Sofat N. Annals Rheumatic Diseases, 2017; 76(10):1764-1773
2) Hunter, D.J., Guermazi, A., Lo, G.H., Grainger, A.J., Conaghan, P.G., Boudreau, R.M. and Roemer, F.W. (2011). Evolution of semi-quantitative whole joint assessment of knee OA: MOAKS (MRI Osteoarthritis Knee Score). Osteoarthritis and Cartilage, 19(8), pp.990–1002.

**Bone Marrow Lesion Score BML-Score**	**Grade**
I. Cysts	
None	0
Present (at least 1)	1
II. Fibrosis (thickened fibrotic connective tissue)	
None	0
Present (at least 1 region)	1
III. Blood vessels (no of BVs)	
Normal (0-15)	0
Increased (> 16)	1
IV. Cartilage (new cartilage formation within bone)	
a. Absent	0
b. Present	1
V. Trabeculae % thickened	
a. Normal (no change)	0
b. Increased thickness	1
VI. Tidemark Integrity	
a. Intact	0
b. Crossed by blood vessels	1
VII. Inflammation (cellular infiltrates)	
a. Absent	0
b. Present	1
**Total**	7

ROI

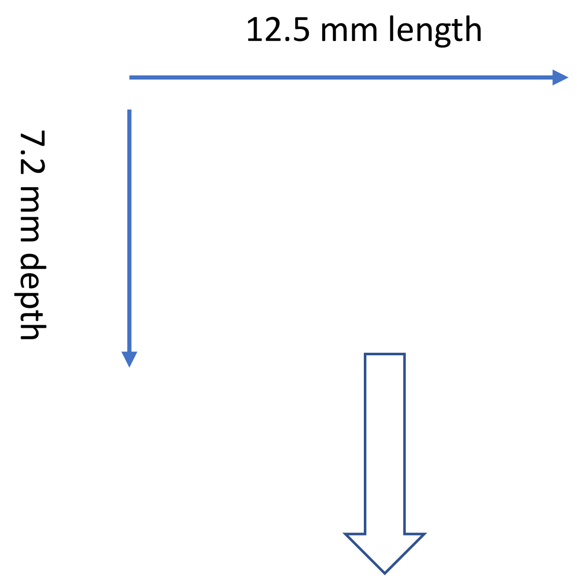

ROI was calculated by taking the MEAN length and depth of all tissues.

Domain 1

CYST

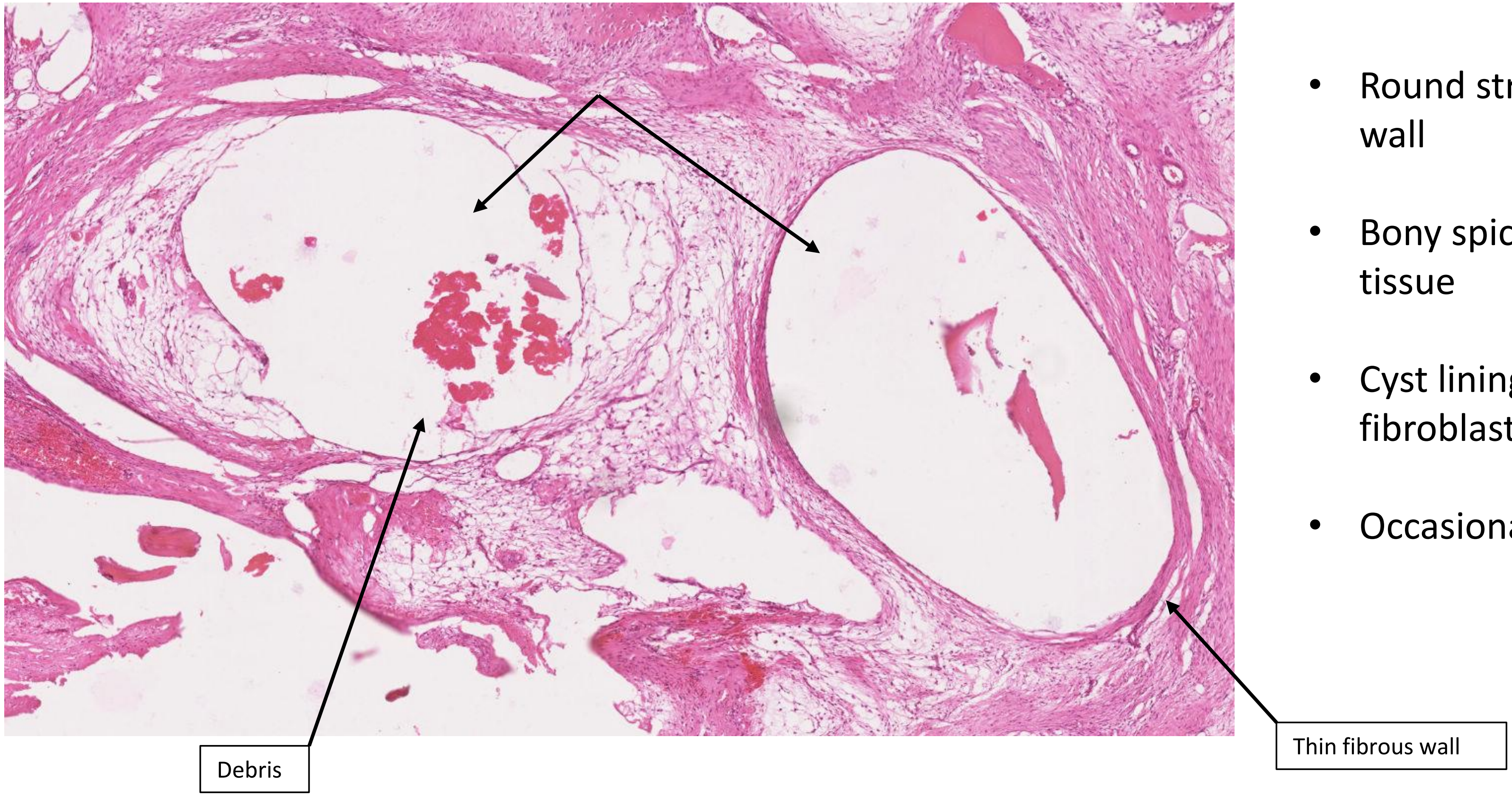

- Round structure with a thin fibrous wall
- Bony spicules replaced by fibrous tissue
- Cyst lining contains flattened fibroblasts
- Occasionally can contain debris

Example of cysts

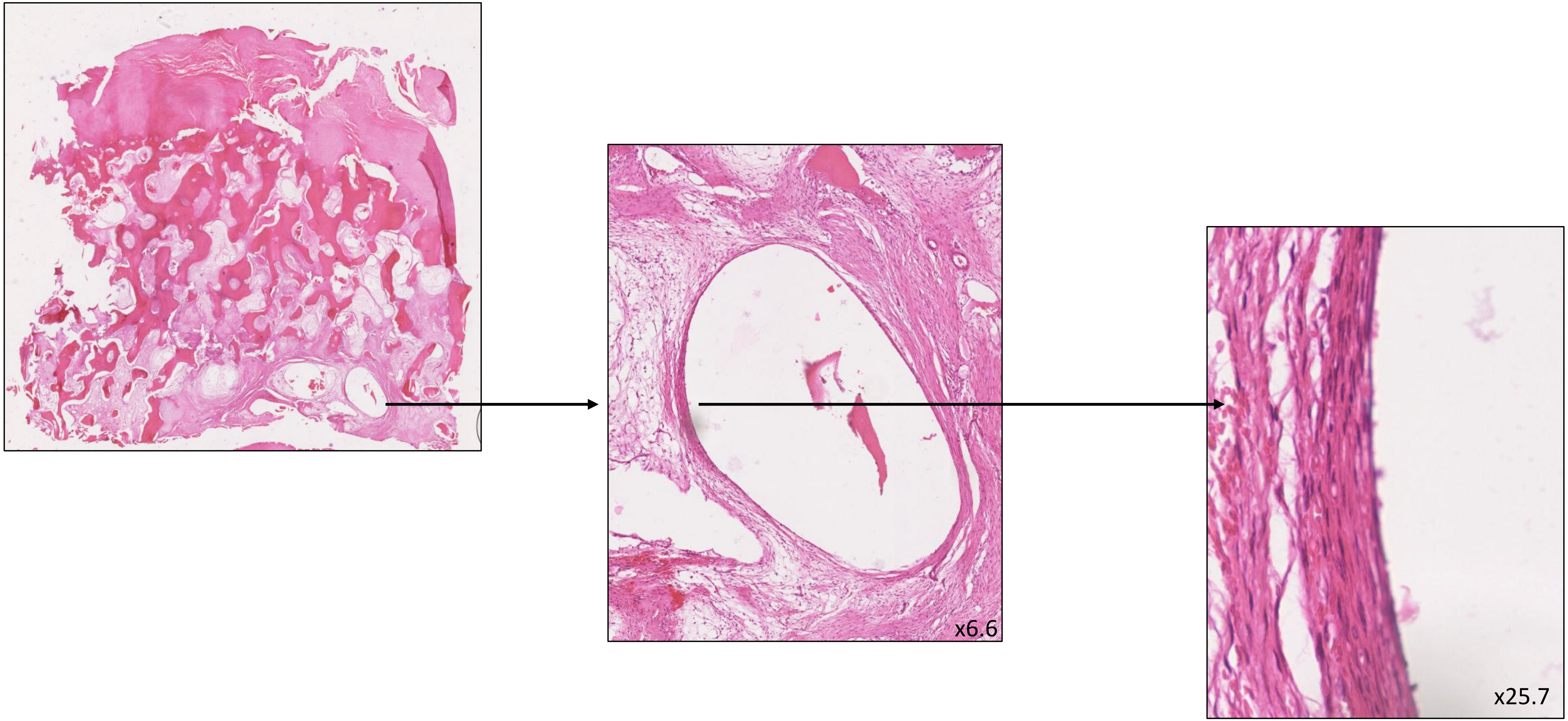

Flattened fibroblasts around lining

Example of cysts

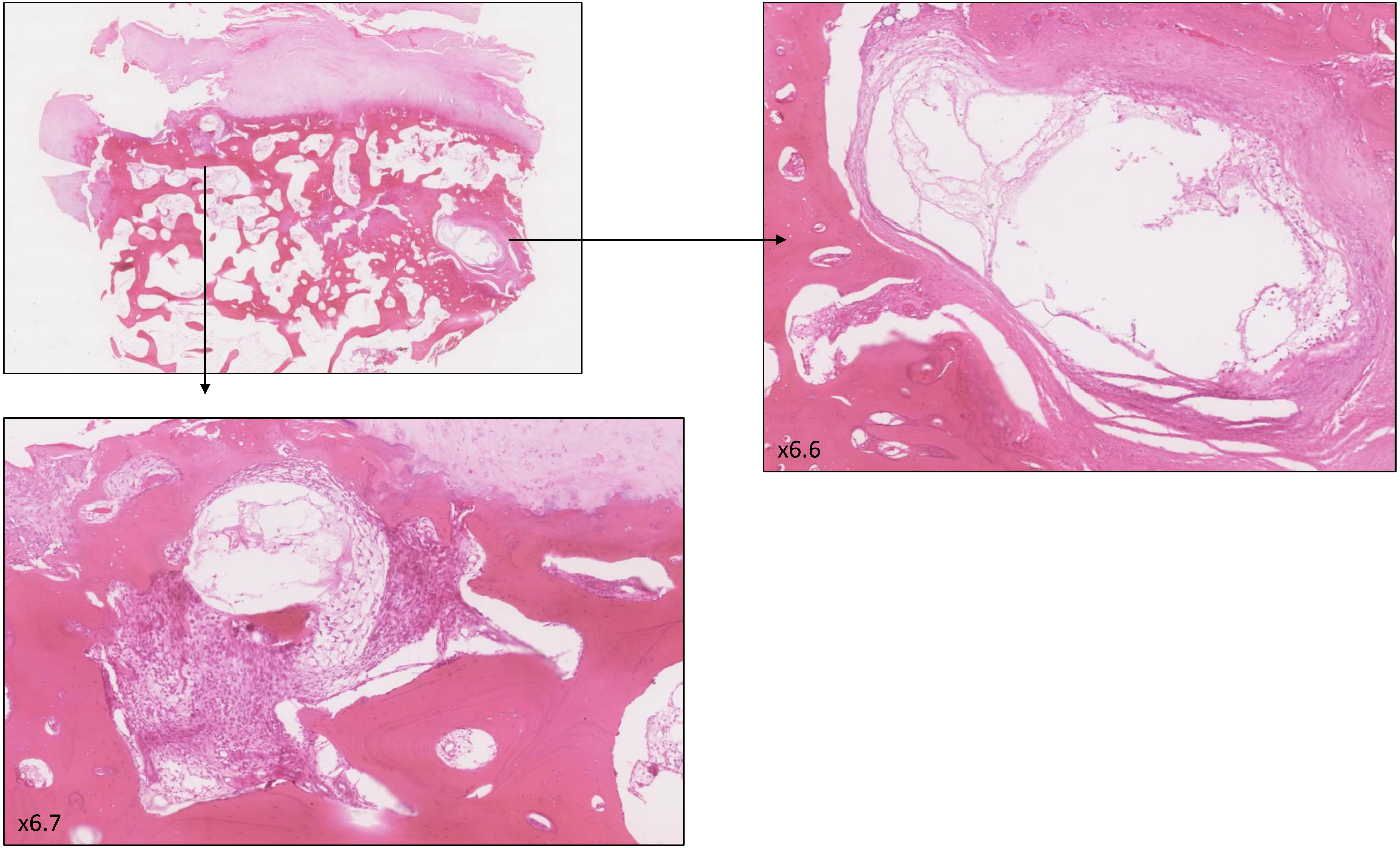

Domain 2

FIBROSIS

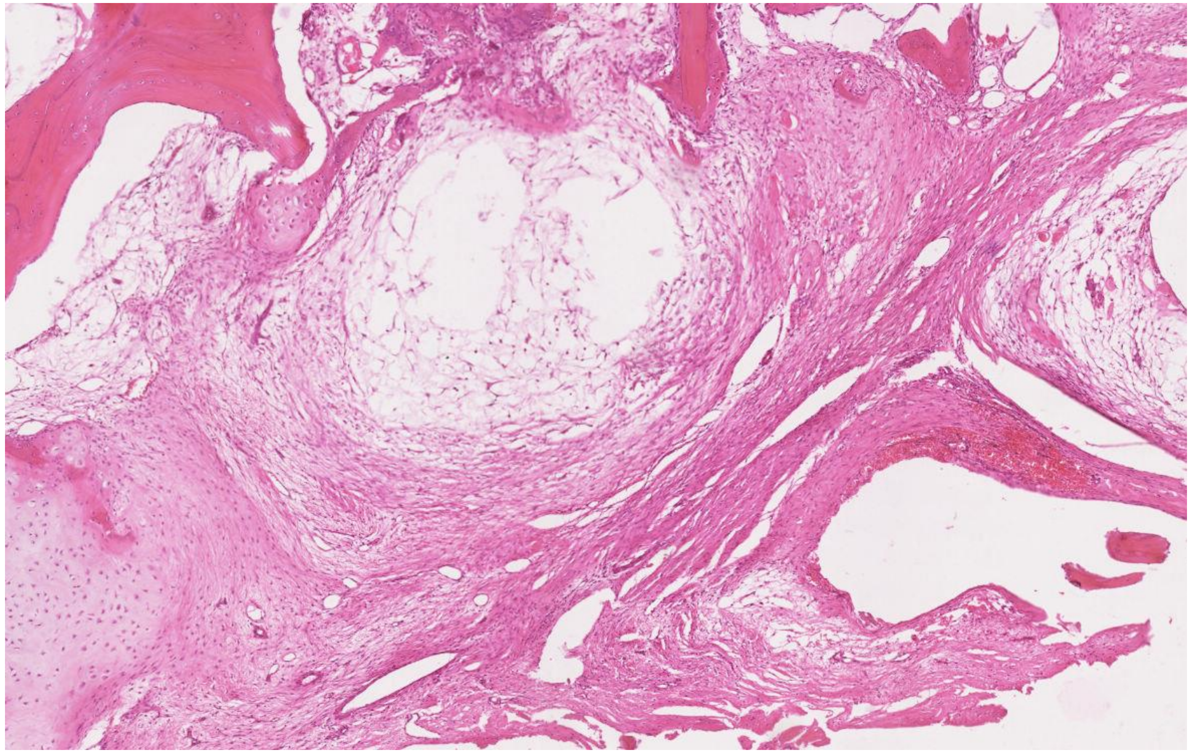

Adipose tissue partially replaced with fibrotic tissue

Sometimes found adjacent the <u>cystic regions</u>

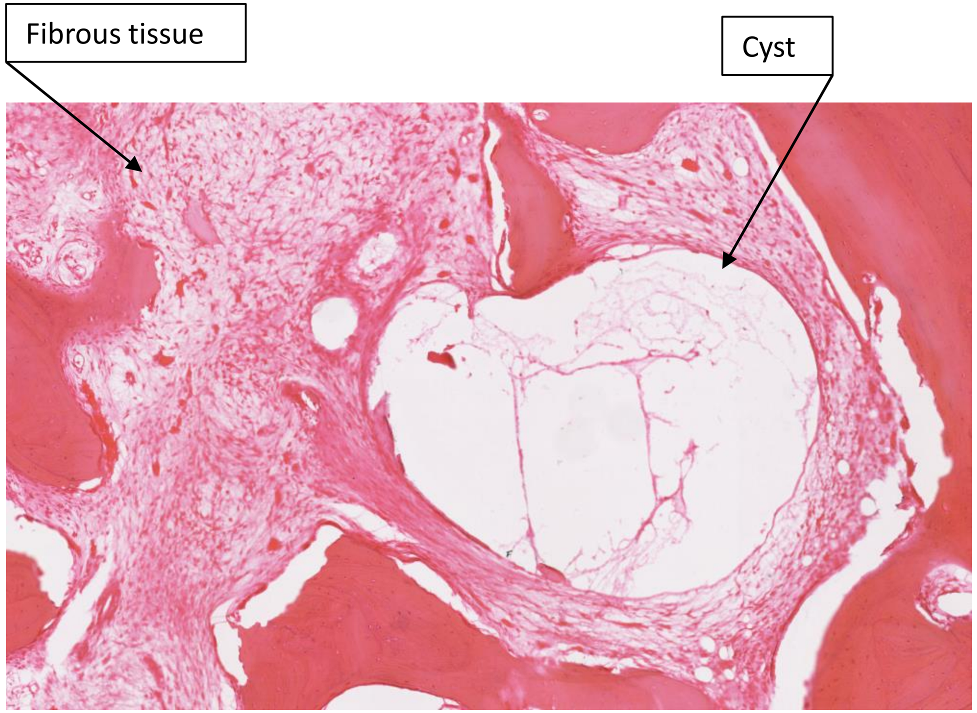

Intensively stained by eosin due to connective tissue fibers.

Domain 3

BLOOD VESSELS

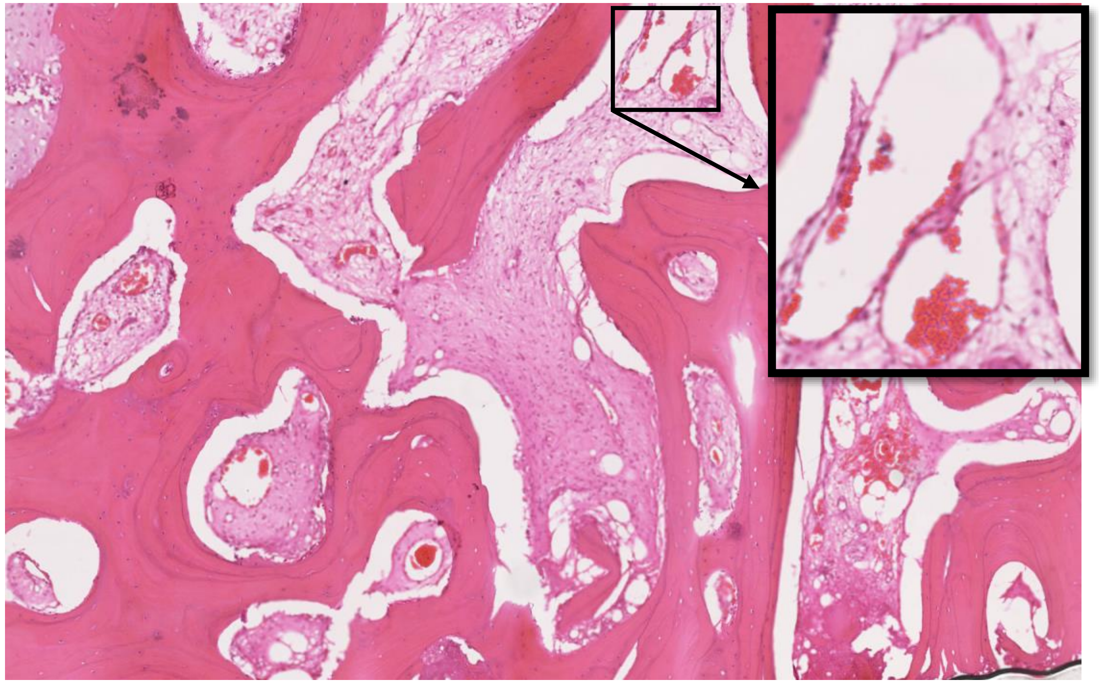

ABNORMAL
Angiogenesis, increased number of blood vessels (>16)

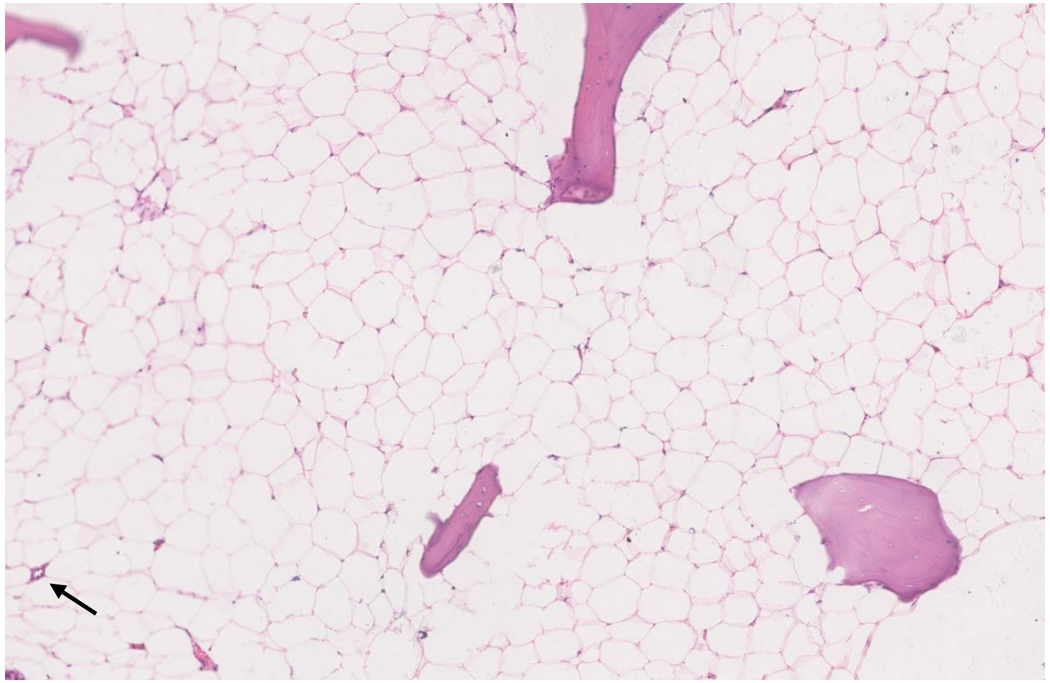

NORMAL
Blood vessels no: 0-15

Domain 4

DE NOVO CARTILAGE (new cartilage formation)

H&E

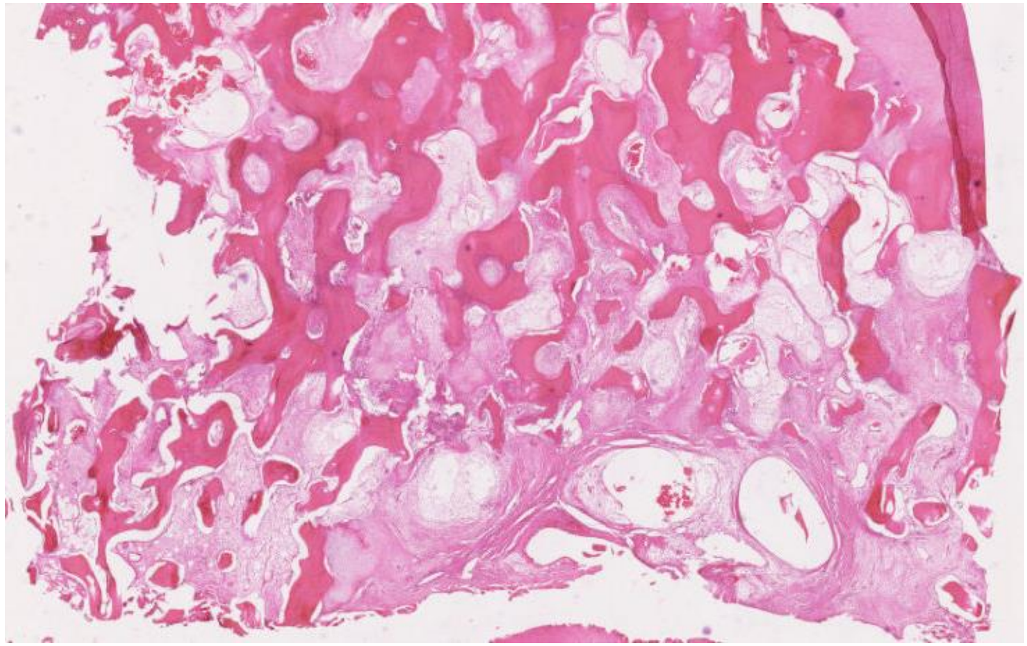

Safranin-0 Fast green

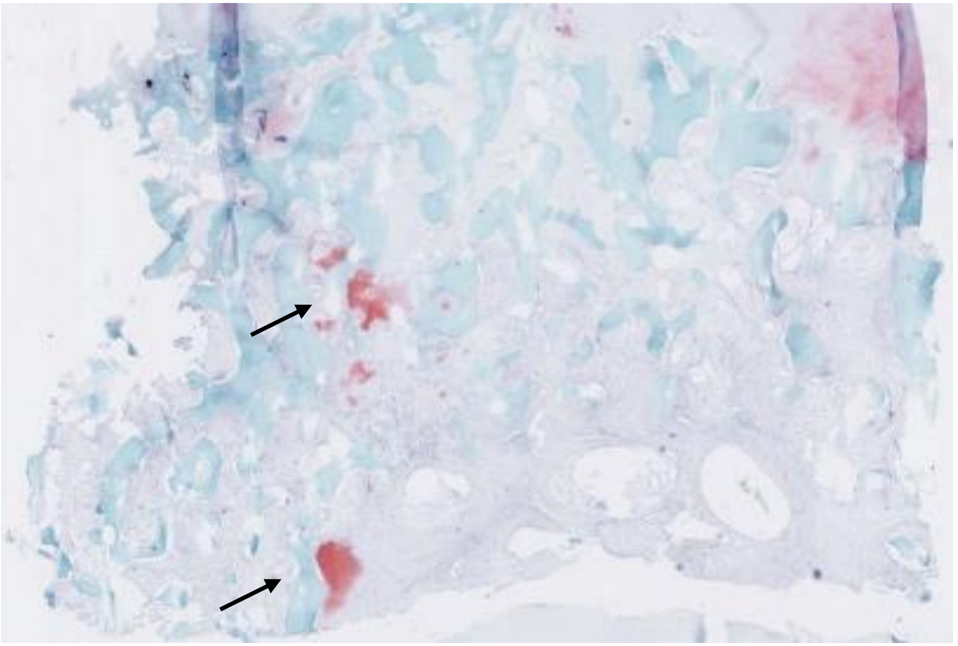

- Presence of new cartilage formation deep in the bone.
- Safranin-O fast green staining required.

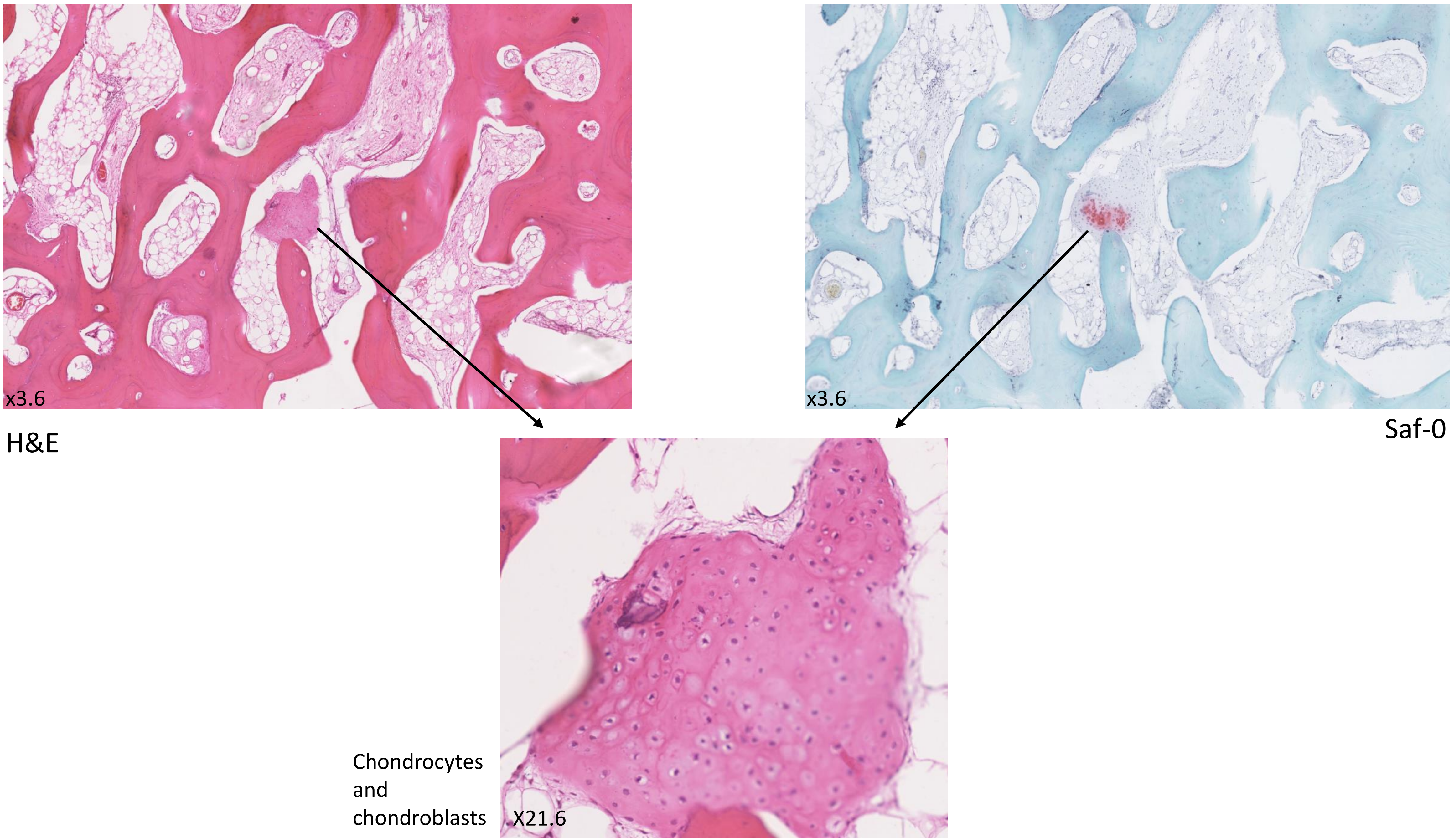
x3.6
H&E
x3.6
Saf-0
Chondrocytes
and
chondroblasts
X21.6

Domain 5

THICKENED TRABECULAE (%)

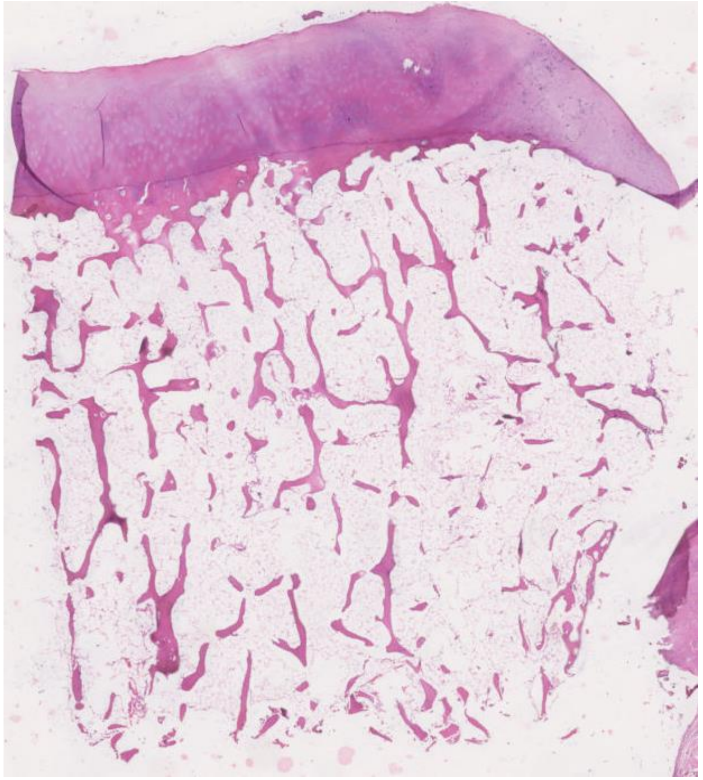

NORMAL

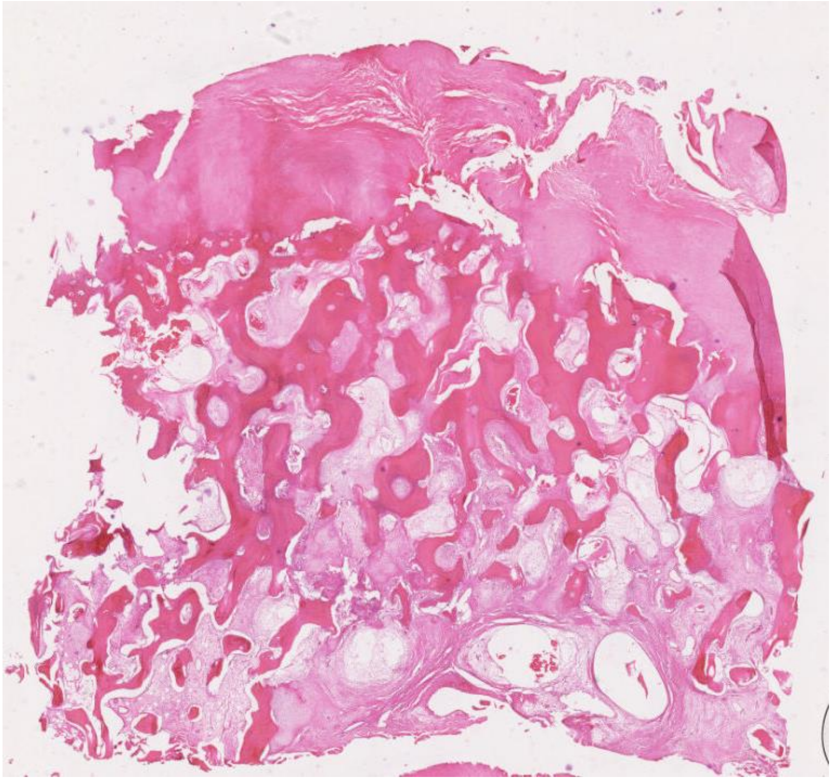

ABNORMAL

- Transformation of trabeculae from rod-like into plate-like
- > 33% of trabeculae thickened (at least).

Domain 6

TIDEMARK INTEGRITY

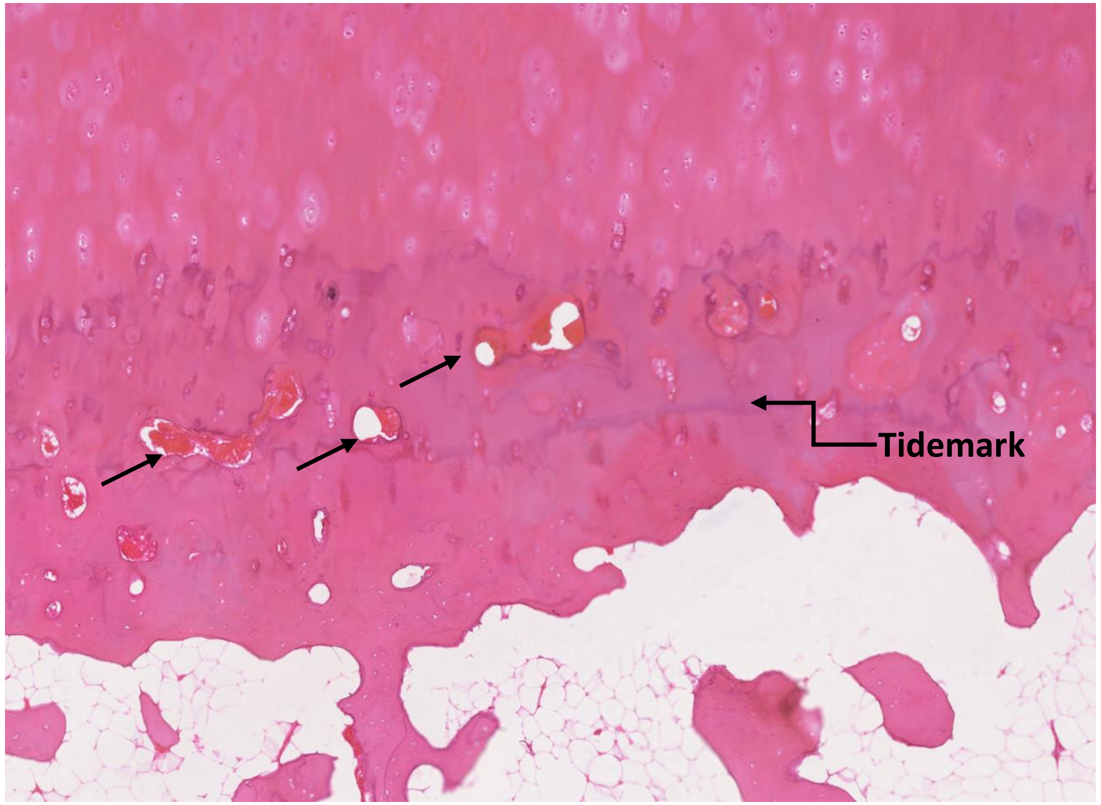

Tidemark breached

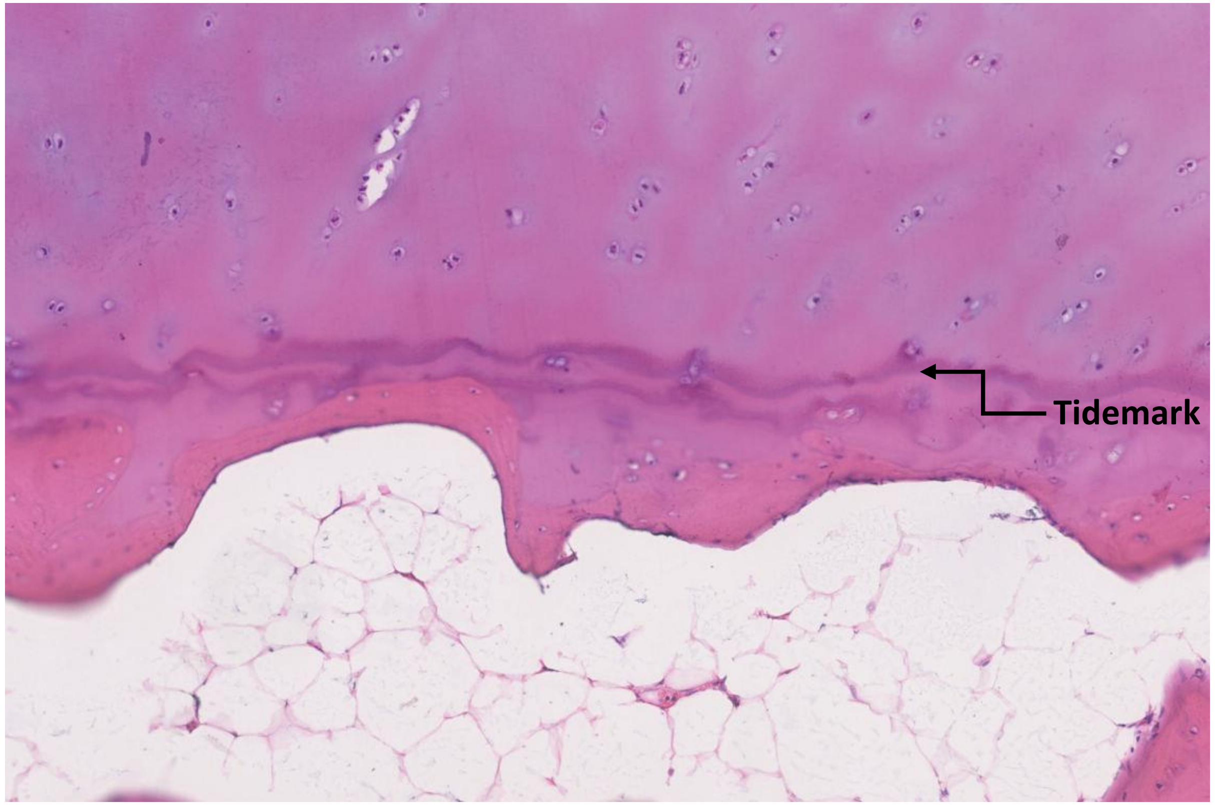

Tidemark NOT breached

- Extension of subchondral blood vessel through the tidemark
- At least 1 BV near OC junction penetrating tidemark

Examples of tidemark integrity

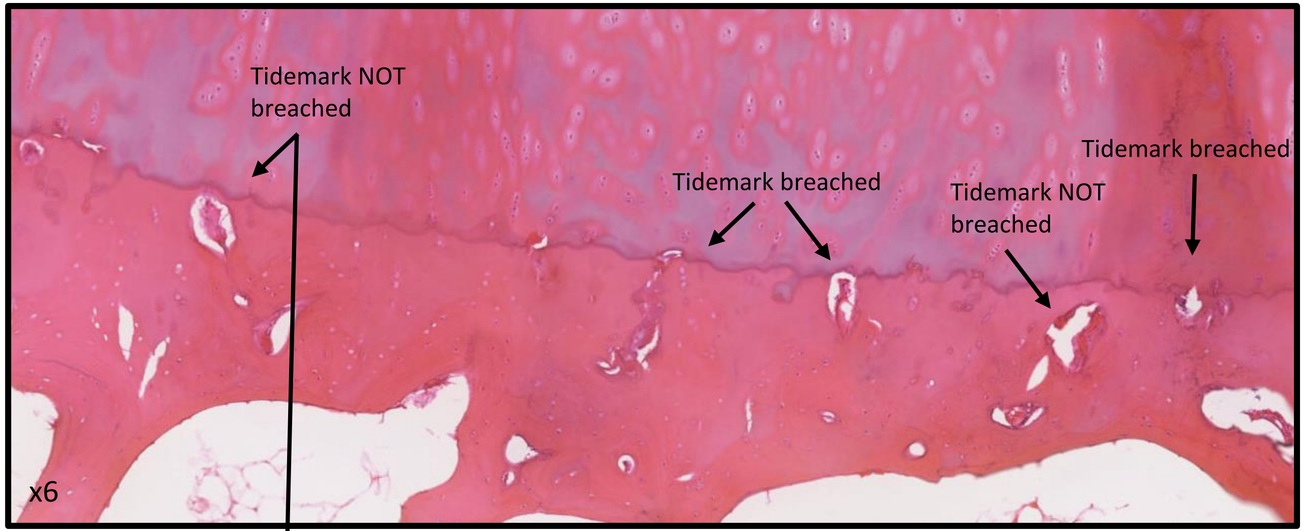

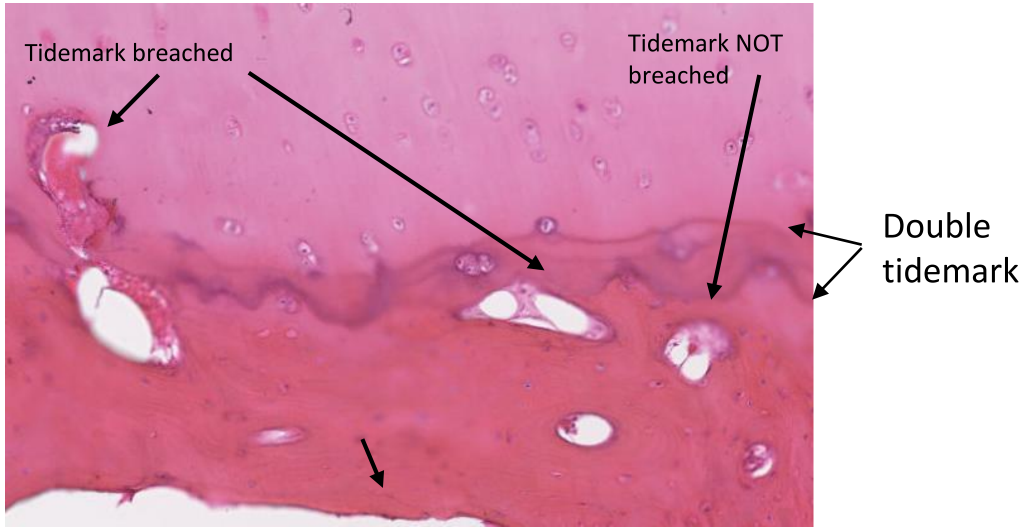

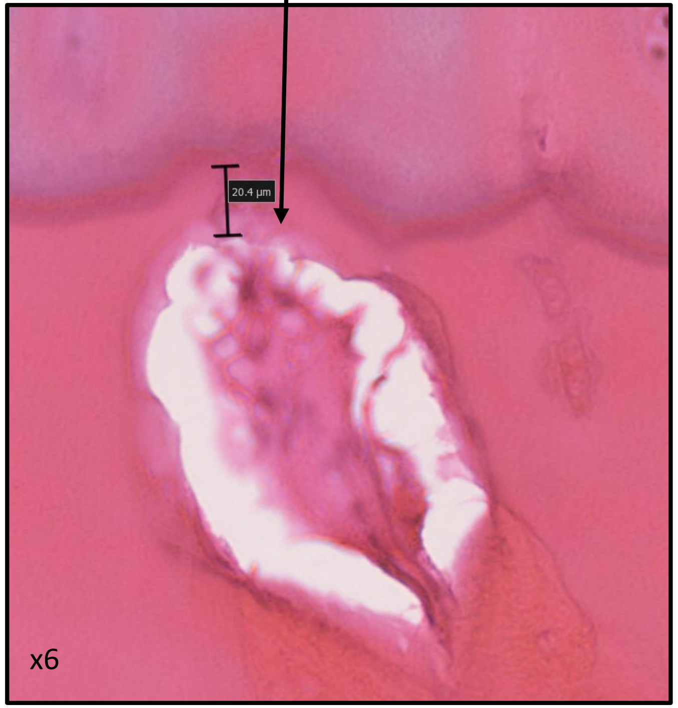

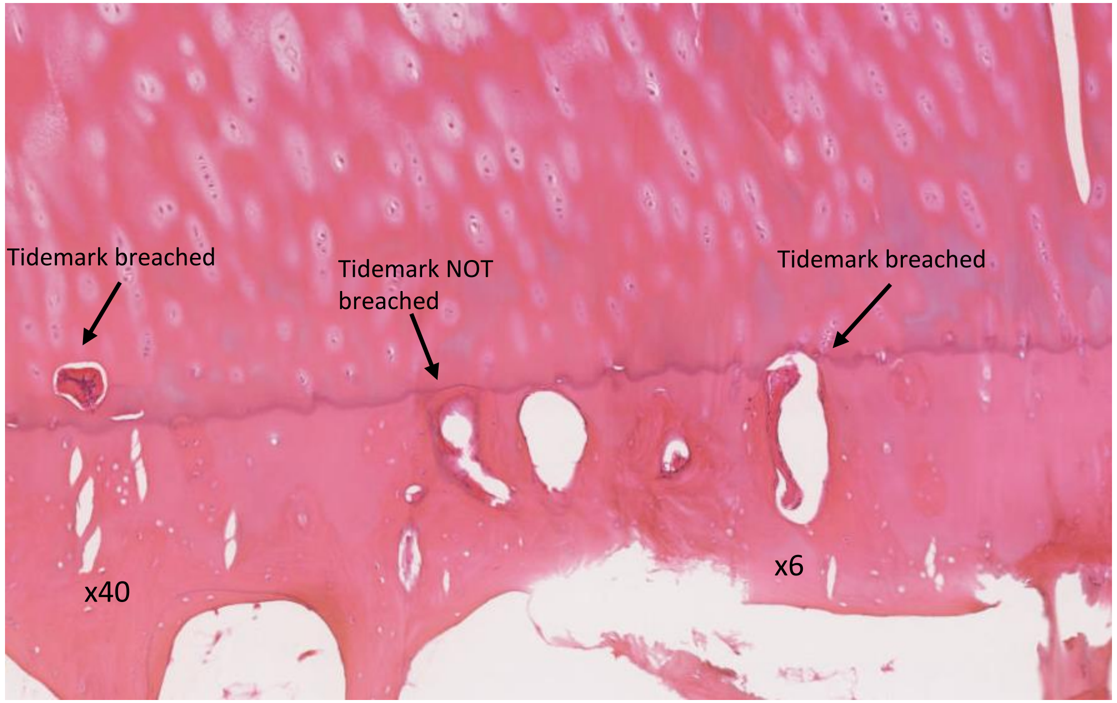

- If BV is touching the tidemark = **breached**
- If just below the tidemark (not touching) = **not breached**
- Ignore double tidemark, tidemark counts as breached if BV touches/breaches first tidemark.

Domain 6

INFLAMMATION

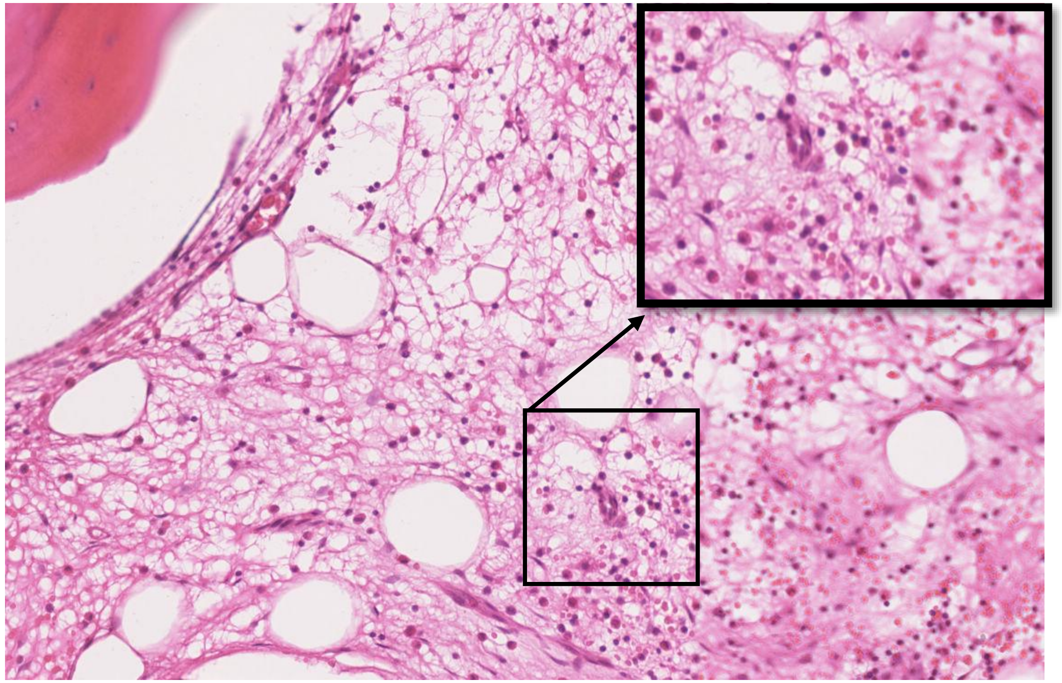

X 14.8

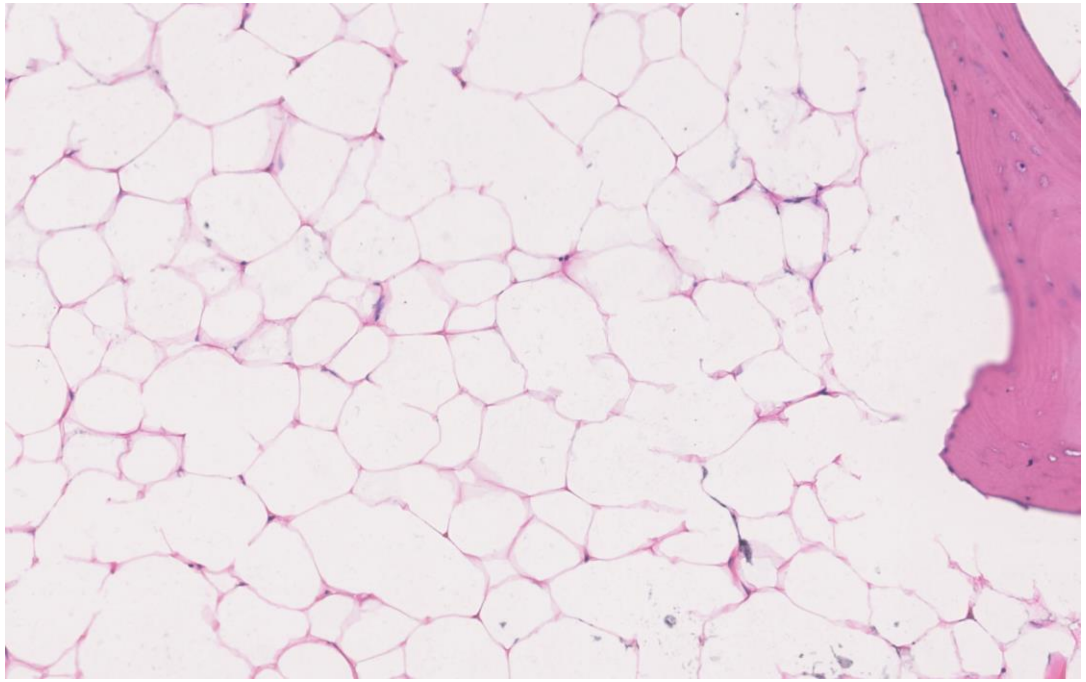

X 14.8

- Cellular infiltrates suggestive of inflammation - any of the following cells (does not have to be all cell types):
- macrophages, granulocytes, lymphocytes, osteoclasts.

Below the tissues we have scored

NEXT

	Grade
Cyst	0
Fibrosis	0
Blood vessels (>15)	1
De novo cartilage	0
Thickened trabeculae	0
Tidemark integrity	1
Inflammation	0
Total	2

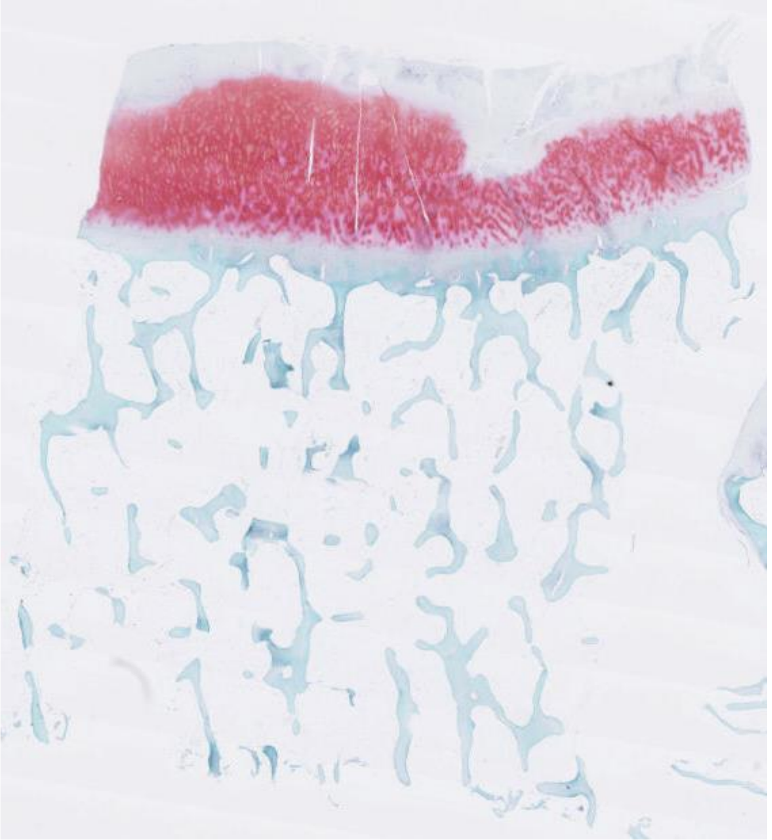

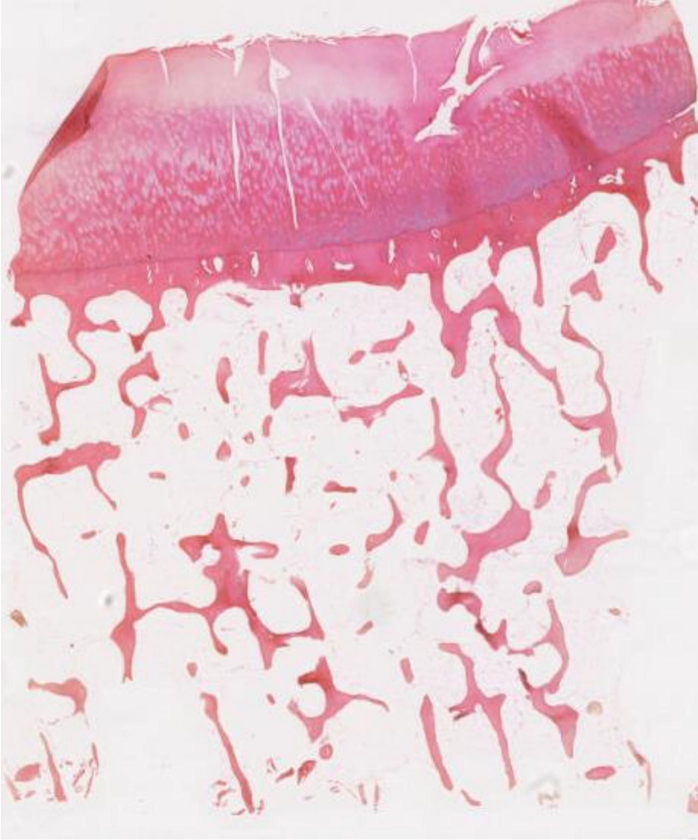

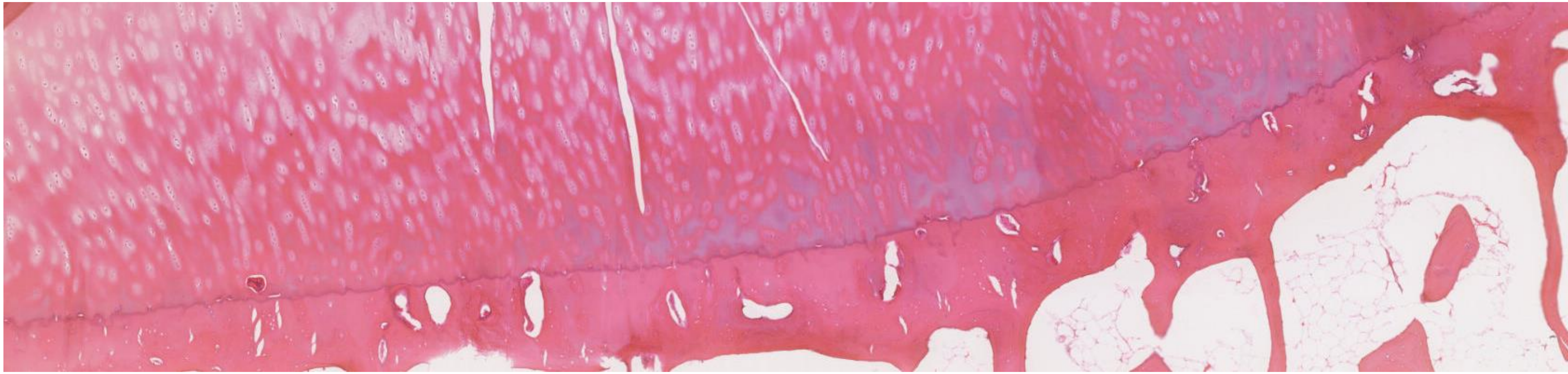

	Grade
Cyst	1
Fibrosis	1
Blood vessels (>15)	1
De novo cartilage	1
Thickened trabeculae	1
Tidemark integrity	1
Inflammation	1
Total	7

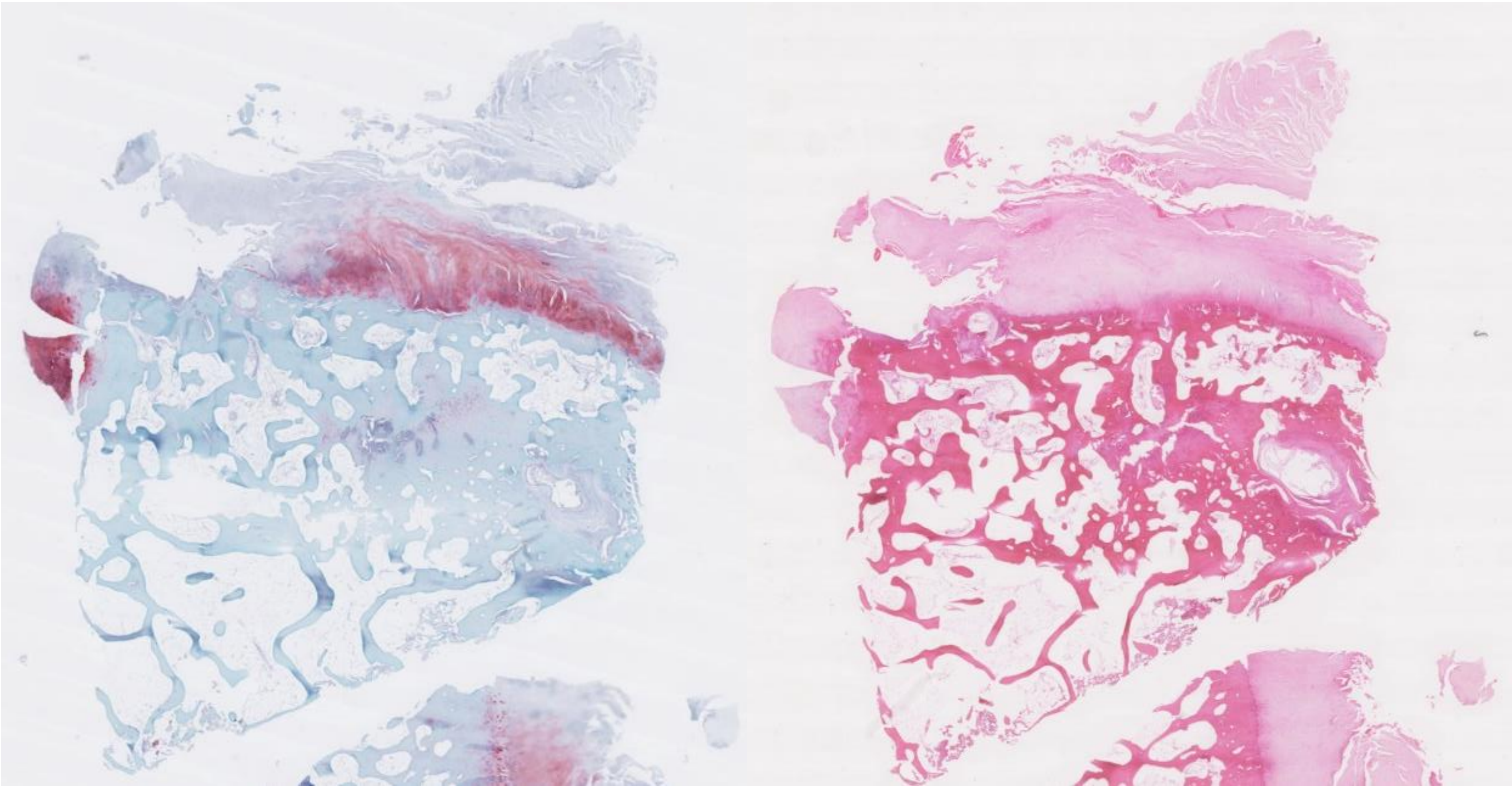

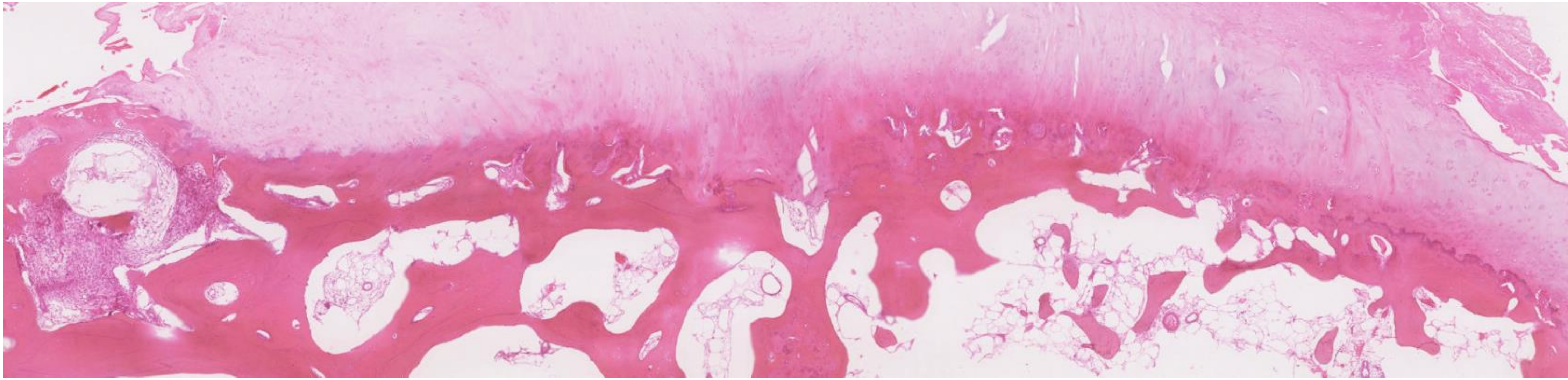

	Grade
Cyst	0
Fibrosis	0
Blood vessels (>15)	0
De novo cartilage	0
Thickened trabeculae	1
Tidemark integrity	1
Inflammation	0
Total	2

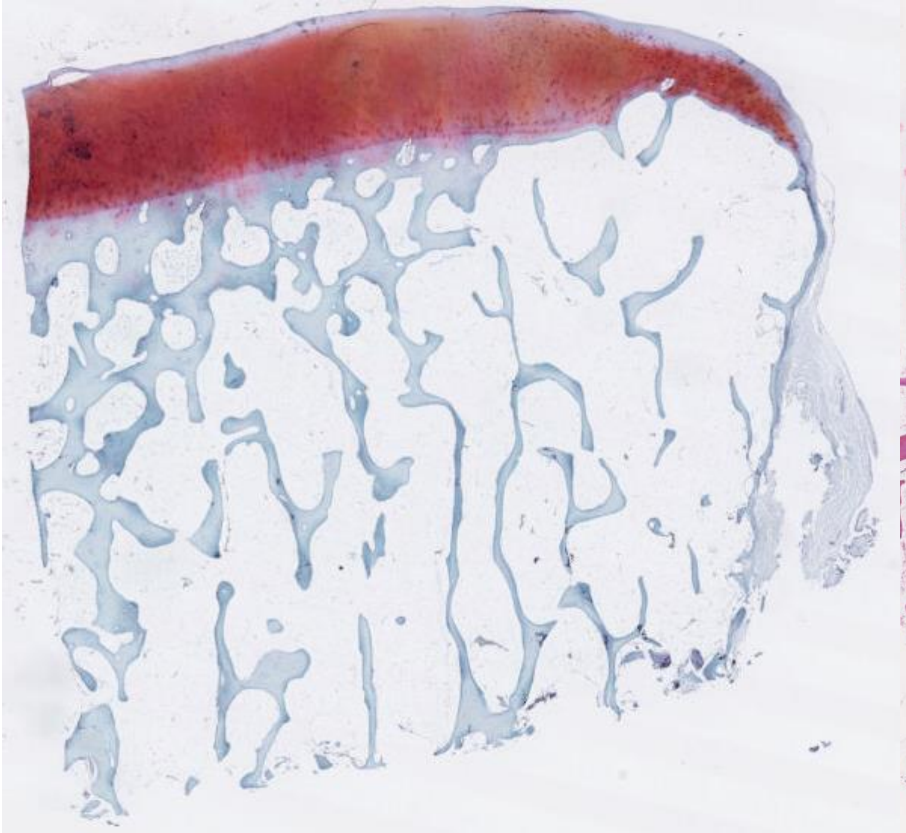

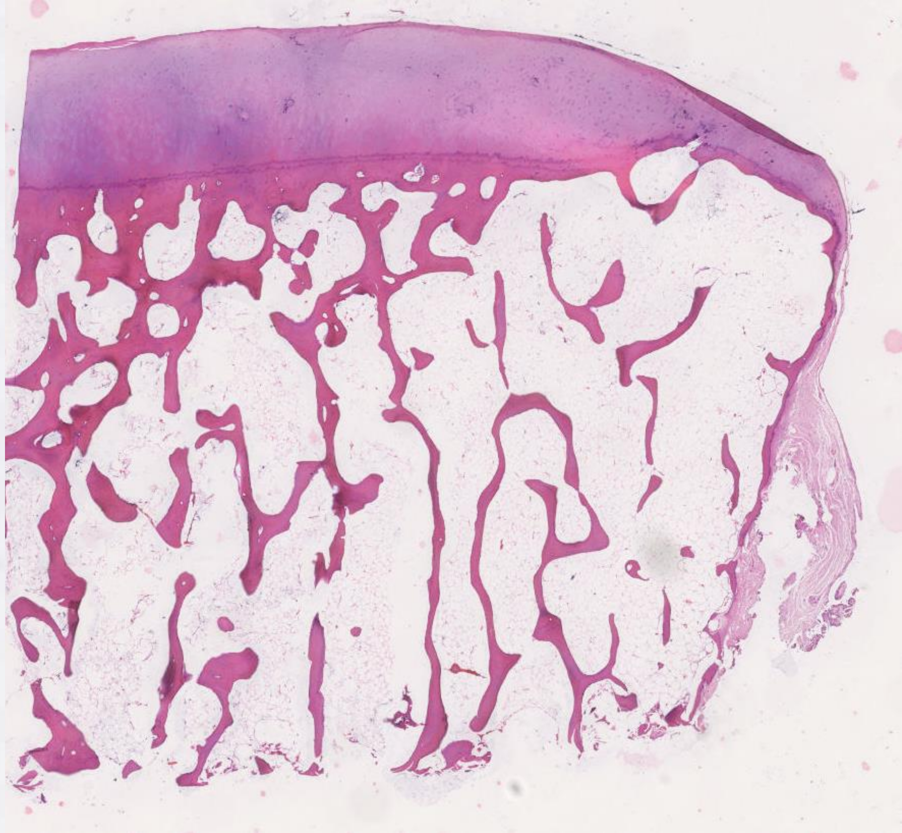

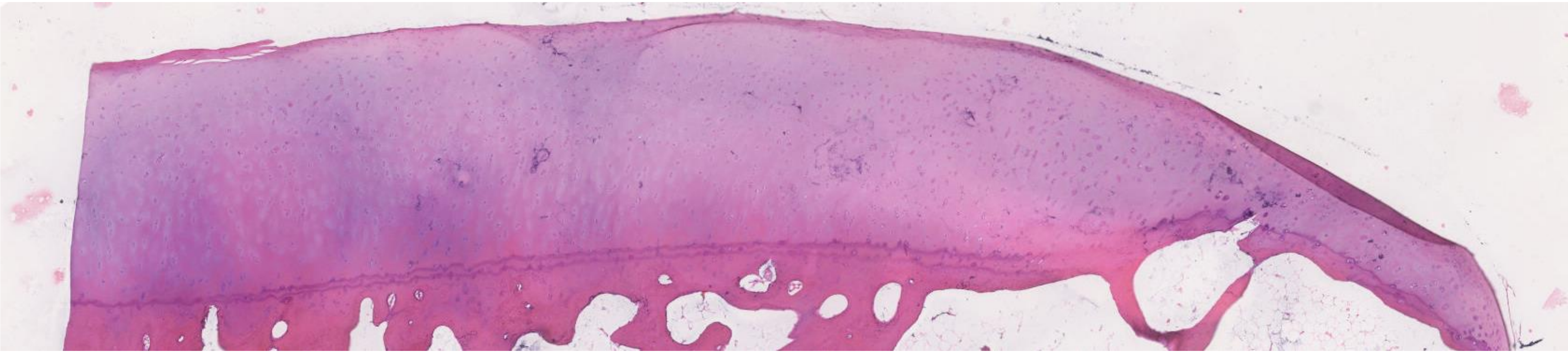

	Grade
Cyst	0
Fibrosis	0
Blood vessels (>15)	0
De novo cartilage	0
Thickened trabeculae	1
Tidemark integrity	1
Inflammation	0
Total	2

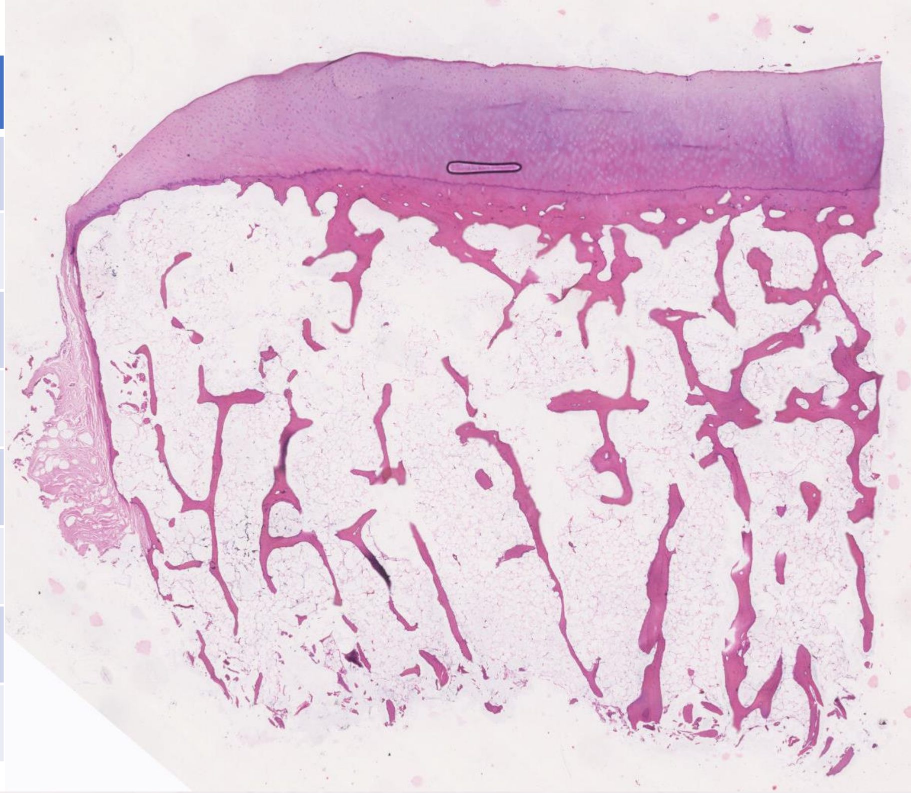

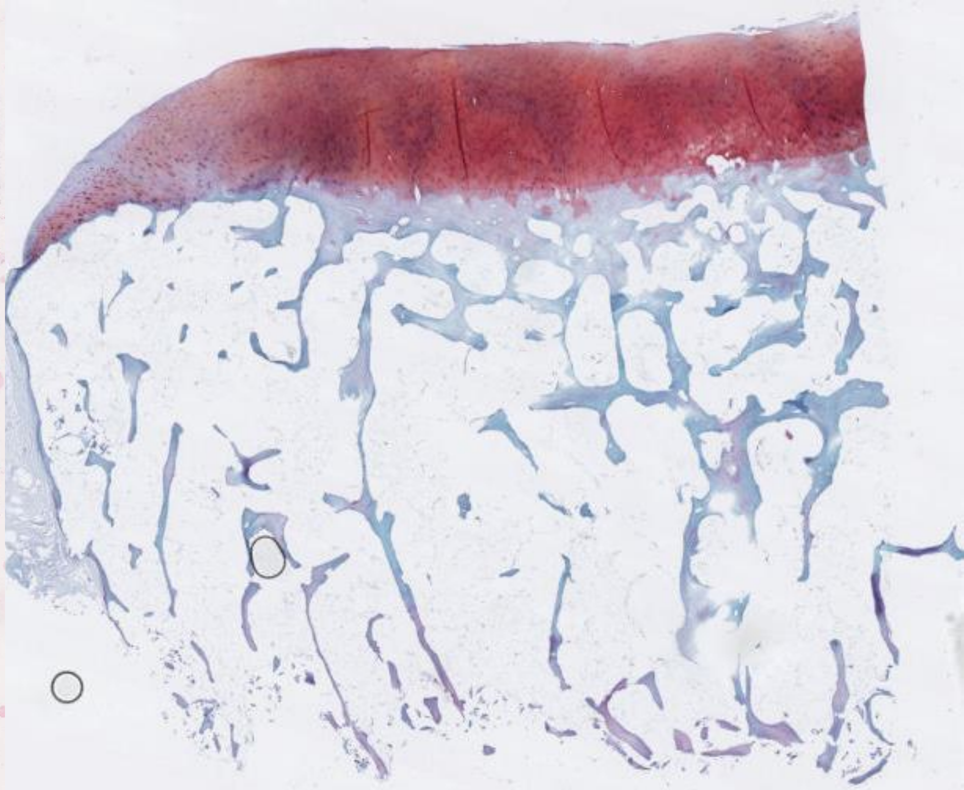

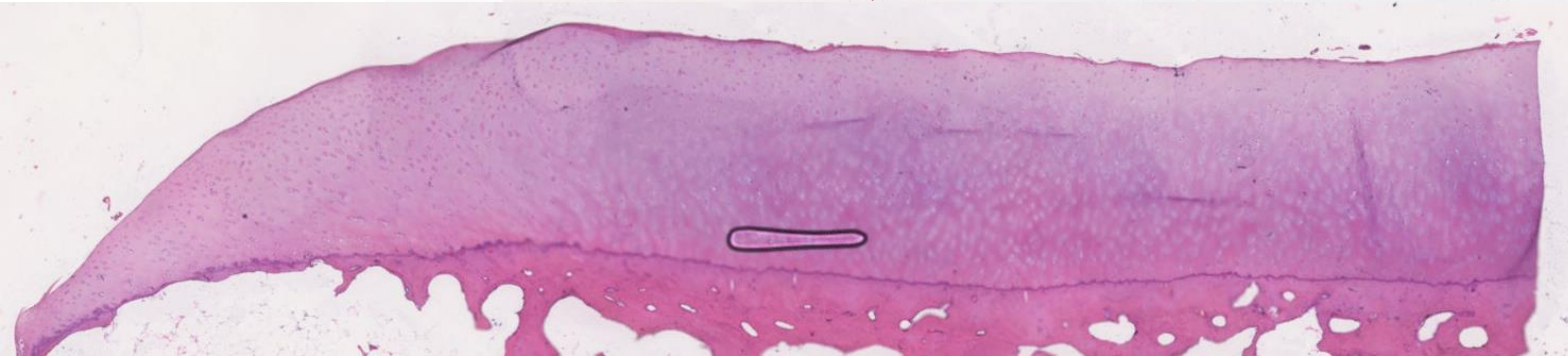

	Grade
Cyst	1
Fibrosis	1
Blood vessels (>15)	1
De novo cartilage	1
Thickened trabeculae	1
Tidemark integrity	1
Inflammation	1
Total	7

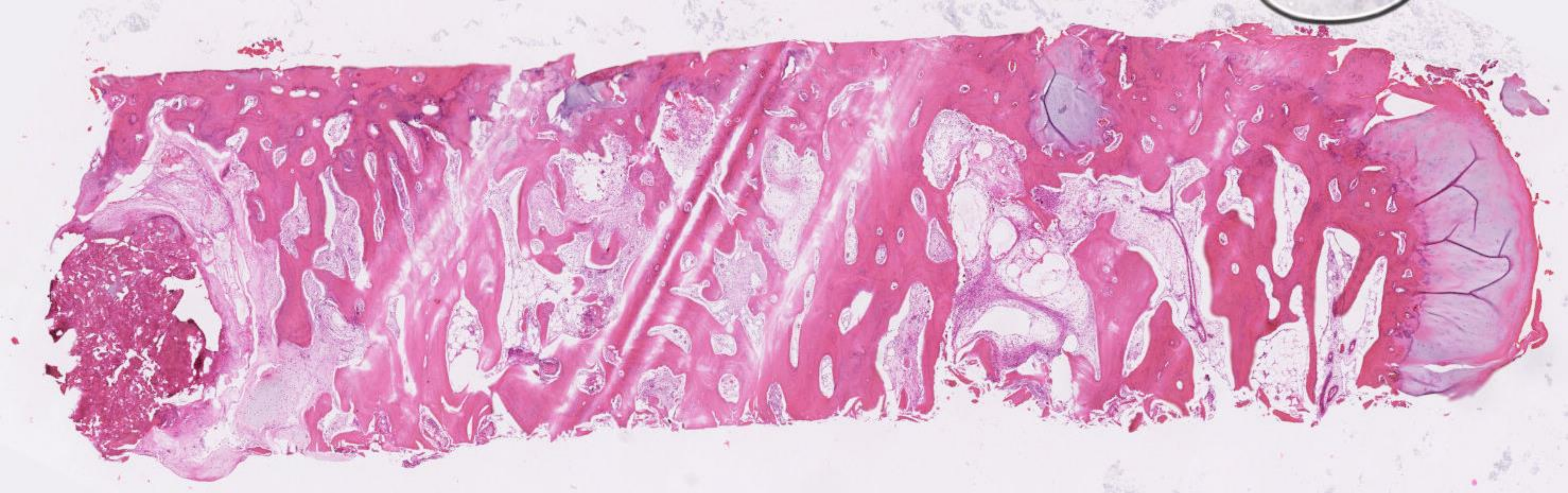

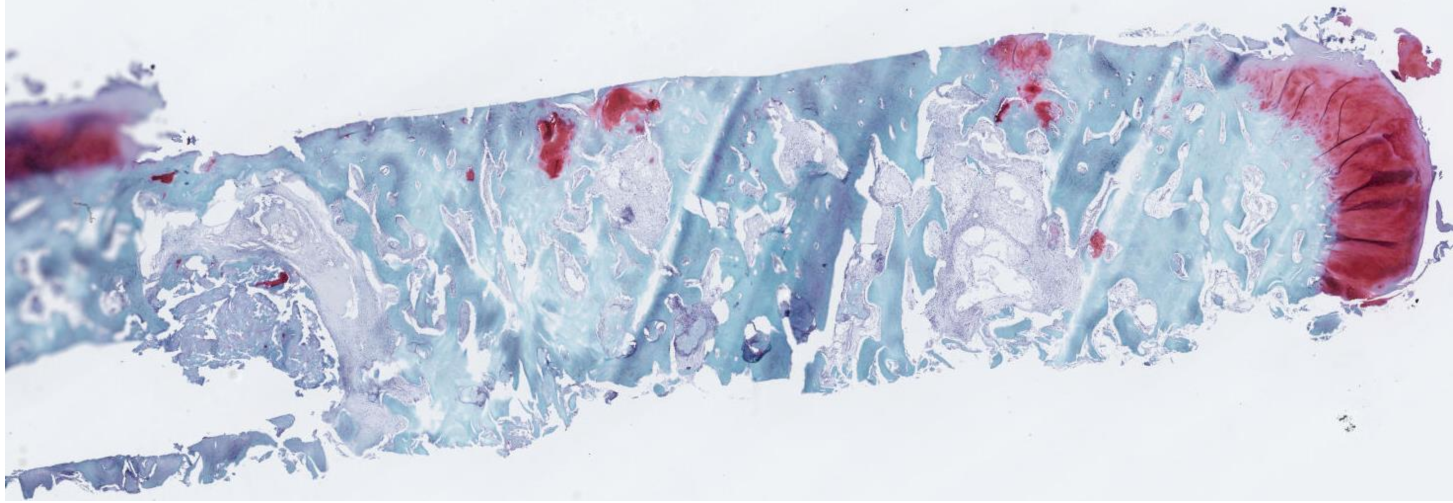

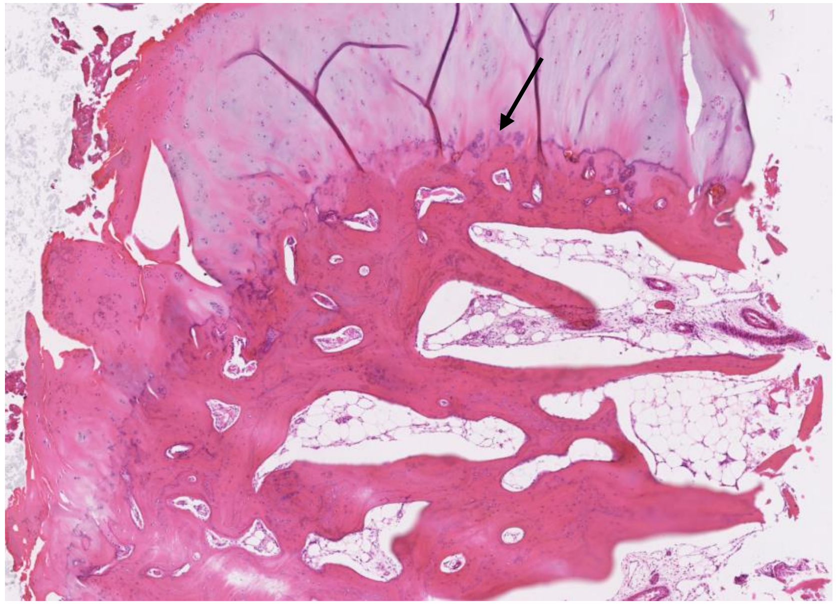

	Grade
Cyst	0
Fibrosis	0
Blood vessels (>15)	1
De novo cartilage	0
Thickened trabeculae	0
Tidemark integrity	1
Inflammation	0
Total	2

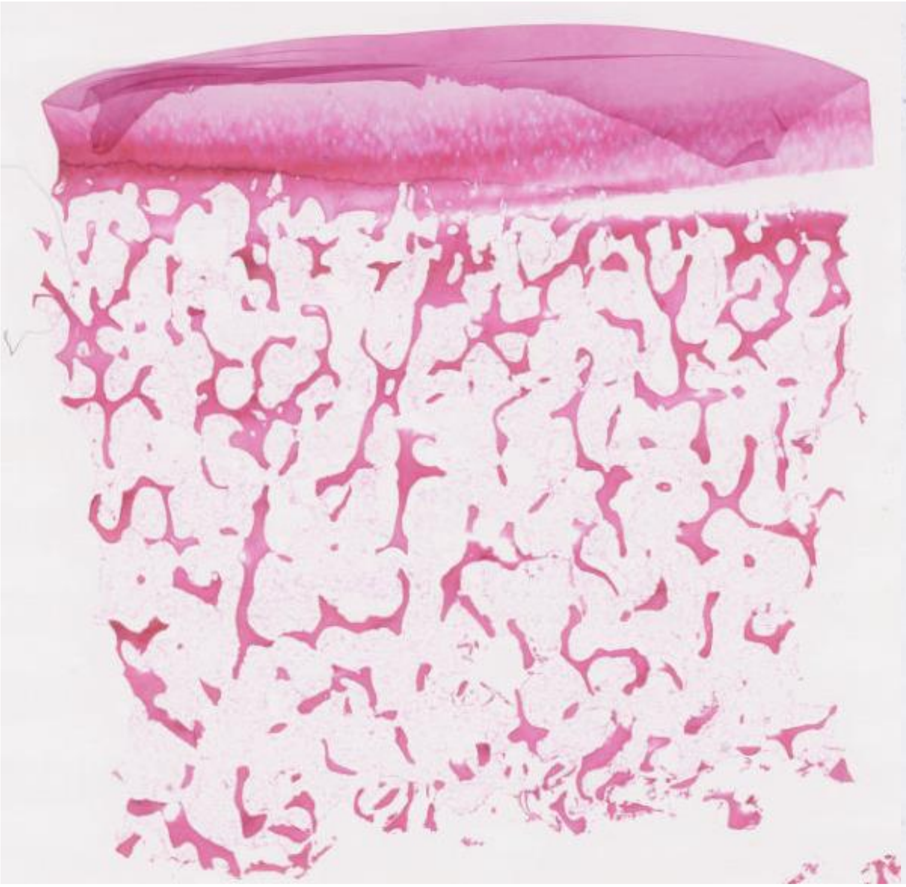

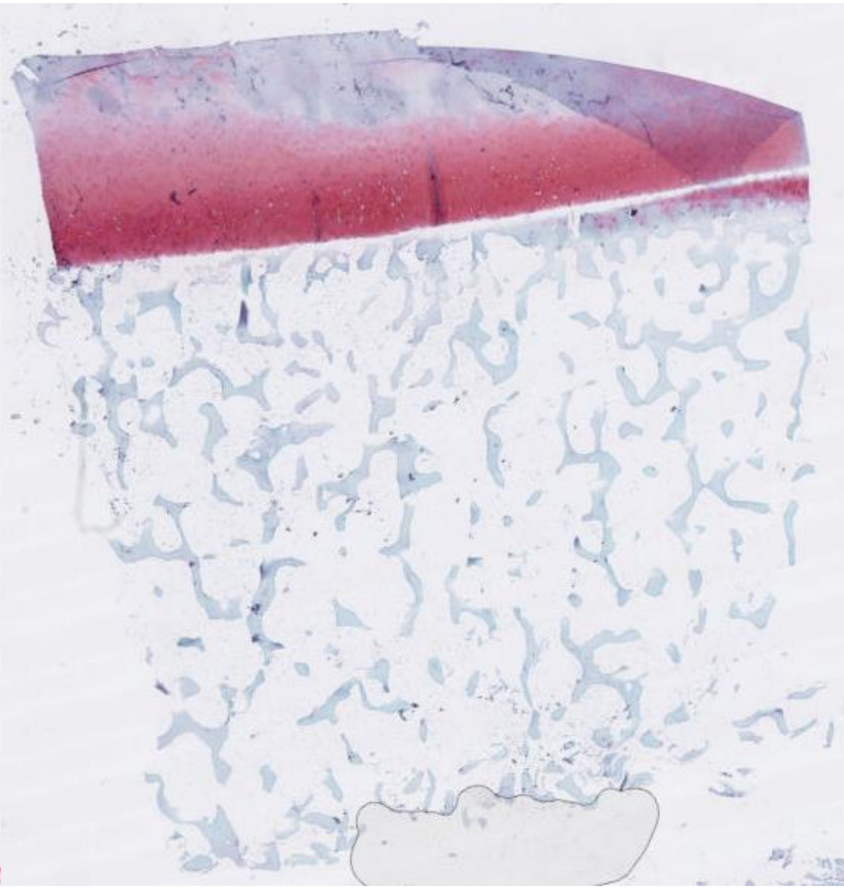

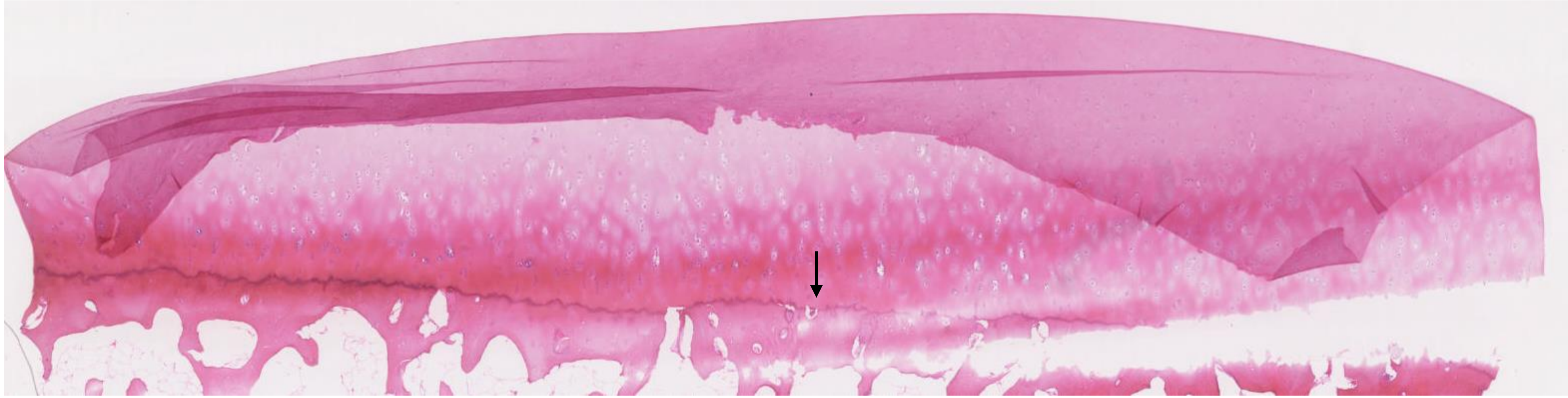

	Grade
Cyst	1
Fibrosis	1
Blood vessels (>15)	1
De novo cartilage	0
Thickened trabeculae	1
Tidemark integrity	1
Inflammation	1
Total	6

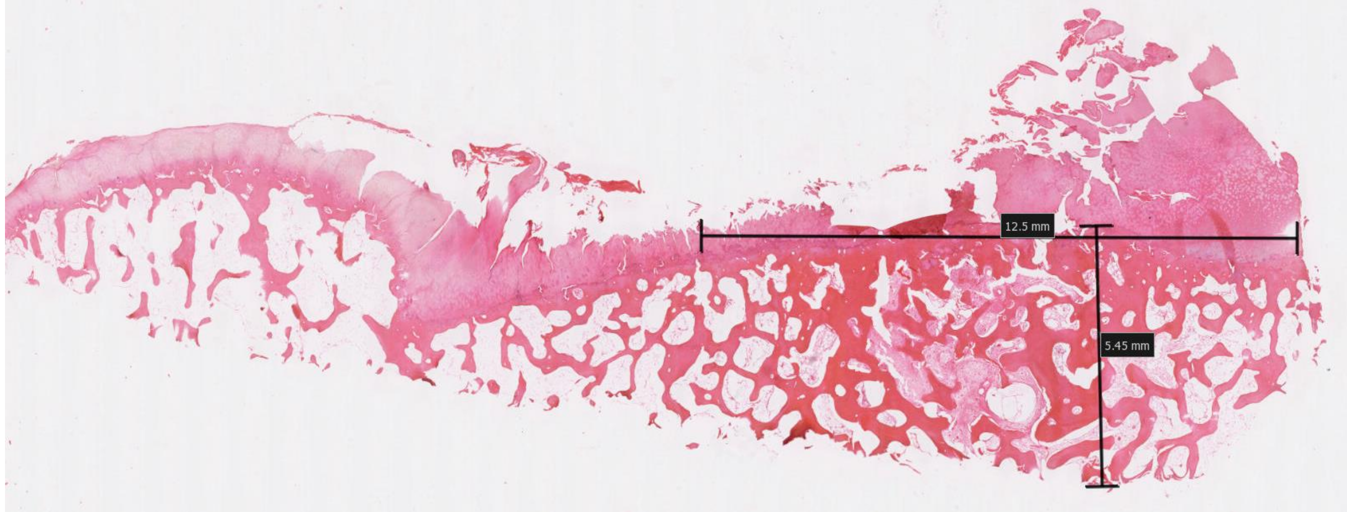

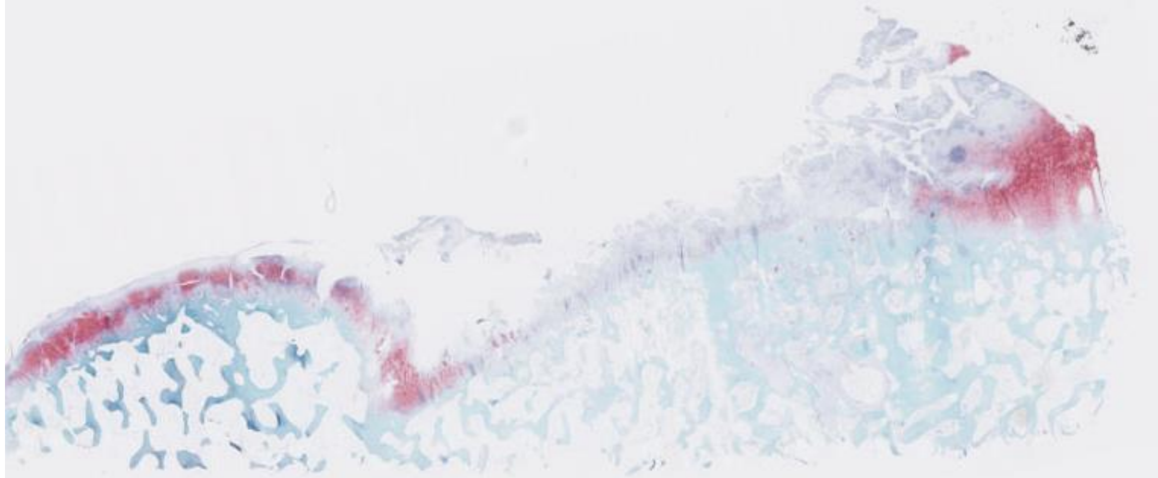

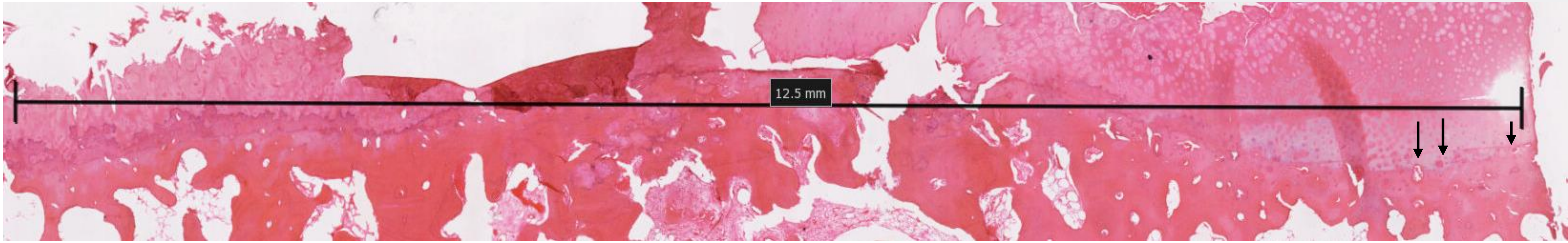

Supplement: Multimedia component 1 [file mmc1.pdf]
